# Regulated RalBP1 Binding to RalA and PSD-95 Controls AMPA Receptor Endocytosis and LTD

**DOI:** 10.1371/journal.pbio.1000187

**Published:** 2009-09-08

**Authors:** Kihoon Han, Myoung-Hwan Kim, Daniel Seeburg, Jinsoo Seo, Chiara Verpelli, Seungnam Han, Hye Sun Chung, Jaewon Ko, Hyun Woo Lee, Karam Kim, Won Do Heo, Tobias Meyer, Hyun Kim, Carlo Sala, Se-Young Choi, Morgan Sheng, Eunjoon Kim

**Affiliations:** 1 National Creative Research Initiative Center for Synaptogenesis and Department of Biological Sciences, Korea Advanced Institute of Science and Technology (KAIST), Daejeon, Korea; 2 The Picower Institute for Learning and Memory, RIKEN-MIT Neuroscience Research Center; 3 Howard Hughes Medical Institute, Massachusetts Institute of Technology, Cambridge, Massachusetts, United States of America; 4 Department of Physiology and Dental Research Institute, Seoul National University School of Dentistry, Seoul, Korea; 5 CNR Institute of Neuroscience and Department of Neurological Sciences, University of Milan, Milan, Italy; 6 Department of Anatomy and Division of Brain Korea 21 Biomedical Science, College of Medicine, Korea University, Seoul, Korea; 7 Department of Chemical and Systems Biology, Stanford University, Stanford, California, United States of America; Duke University Medical Center, United States of America

## Abstract

Long-term depression (LTD) is a long-lasting activity-dependent decrease in synaptic strength. NMDA receptor (NMDAR)–dependent LTD, an extensively studied form of LTD, involves the endocytosis of AMPA receptors (AMPARs) via protein dephosphorylation, but the underlying mechanism has remained unclear. We show here that a regulated interaction of the endocytic adaptor RalBP1 with two synaptic proteins, the small GTPase RalA and the postsynaptic scaffolding protein PSD-95, controls NMDAR-dependent AMPAR endocytosis during LTD. NMDAR activation stimulates RalA, which binds and translocates widespread RalBP1 to synapses. In addition, NMDAR activation dephosphorylates RalBP1, promoting the interaction of RalBP1 with PSD-95. These two regulated interactions are required for NMDAR-dependent AMPAR endocytosis and LTD and are sufficient to induce AMPAR endocytosis in the absence of NMDAR activation. RalA in the basal state, however, maintains surface AMPARs. We propose that NMDAR activation brings RalBP1 close to PSD-95 to promote the interaction of RalBP1-associated endocytic proteins with PSD-95-associated AMPARs. This suggests that scaffolding proteins at specialized cellular junctions can switch their function from maintenance to endocytosis of interacting membrane proteins in a regulated manner.

## Abstract 2

A two step mechanism was identified that regulates receptor endocytosis during the development of long-term depression (LTD), a long-lasting decrease in synaptic transmission.

## Author Summary

Neurons adapt over time in order to dampen their response to prolonged or particularly strong stimuli. This process, termed long-term depression (LTD), results in a long-lasting decrease in the efficiency of synaptic transmission. One extensively studied form of LTD requires the activation of a specific class of receptors known as NMDA glutamate receptors (NMDARs). A key molecular event initiated by NMDA receptor activation is the stimulation of protein phosphatases. Another key event is internalization via endocytosis of synaptic AMPA glutamate receptors (AMPARs). However, the mechanism by which protein dephosphorylation is coupled to AMPAR endocytosis has remained unclear. Here, we help to define this mechanism. We show that endocytic proteins, including RalBP1, are widely distributed in neurons under normal conditions. Upon NMDAR activation, the small GTPase RalA becomes activated and binds to RalBP1, resulting in the translocation of RalBP1 and RalBP1-associated endocytic proteins to synapses. At the same time, RalBP1 becomes dephosphorylated, which promotes its binding to the postsynaptic scaffold protein PSD-95, a protein that itself associates with AMPARs. This concerted recruitment of endocytic proteins to the vicinity of AMPARs results in AMPAR endocytosis. On the basis of our data, we propose a model in which dual binding of RalBP1 to both RalA and PSD-95 following RalBP1 dephosphorylation is essential for NMDAR-dependent AMPAR endocytosis during LTD.

## Introduction

Long-term depression (LTD), a long-lasting activity-dependent decrease in synaptic strength, has been implicated in brain development, learning and memory, drug addiction, and mental retardation [1]. NMDA receptor (NMDAR)–dependent LTD, an extensively studied form of LTD, involves a rise in postsynaptic Ca^2+^ concentration, activation of a serine-threonine protein phosphatase cascade, and clathrin-dependent rapid endocytosis of AMPA receptors (AMPARs) [1]–[10]. However, little is known about the identity of key phosphatase substrates and, more importantly, how their dephosphorylation promotes AMPAR endocytosis during NMDAR-dependent LTD. Previous studies have identified several phosphatase substrates associated with NMDAR-dependent LTD and AMPAR endocytosis, including the GluR1 subunit of AMPARs [11]–[13], stargazin [14], and PSD-95 [15]. However, their dephosphorylation has not been clearly linked to the regulation of AMPAR endocytosis. In addition, the clathrin adaptor complex AP2, which directly binds to AMPARs and is required for NMDAR-dependent AMPAR endocytosis and LTD [5],[16], should be brought close to AMPARs in a tightly regulated manner, but little is known about the underlying mechanism.

AMPA receptors have two distinct auxiliary subunits, TARP/stargazin and cornichons, that regulate AMPAR trafficking and gating [17],[18]. Of these, TARP/stargazin is known to anchor AMPARs to synapses by directly interacting with PSD-95, an abundant postsynaptic scaffolding protein implicated in the regulation of excitatory synaptic structure, function, and plasticity [19]–[21]. Accordingly, PSD-95 is a key determinant of synaptic levels of AMPARs [21],[22]. PSD-95, as a protein that is directly coupled to the AMPAR/TARP complex, is in an ideal position to determine or regulate the fate of AMPARs during their activity-dependent trafficking. Indeed, mice null for PSD-95 and those carrying truncated PSD-95 show markedly enhanced LTP [23],[24]. In addition, acute knockdown of PSD-95 in brain slices impairs LTD [25],[26]. These results suggest that PSD-95 is important for the regulation of synaptic plasticity, but the underlying molecular mechanisms are only just beginning to be understood [15],[26]–[28].

The RalA small GTPase is an important regulator of bidirectional membrane traffic (endocytosis and exocytosis) and regulates diverse biological processes, including cell migration, apoptosis, transcription, proliferation, differentiation, and oncogenesis [29],[30]. Whether RalA promotes endocytosis or exocytosis depends on RalA interaction with downstream effectors. Endocytosis occurs when active RalA associates with RalBP1/RLIP76 [31],[32], an endocytic adaptor that directly interacts with the μ2 subunit of the endocytic AP2 complex [33] and EH domain proteins POB1 and Reps1 [34],[35]. RalA promotes exocytosis when it associates with Sec5 and Exo84 subunits of the exocyst complex [29],[30]. However, little is known about how RalA binding to RalBP1 and the exocyst complex is coupled to the endocytosis and exocytosis of specific target membrane proteins, respectively.

We found that NMDAR activation induces RalA activation and that activated RalA binds and translocates RalBP1 to dendritic spines. In addition, NMDAR activation leads to the dephosphorylation of RalBP1, which promotes RalBP1 binding to PSD-95. Our data suggest that these two regulated interactions are required and sufficient for the induction of AMPAR endocytosis during NMDAR-dependent LTD.

## Results

### PSD-95 Interacts with RalBP1

**Figure 1:**
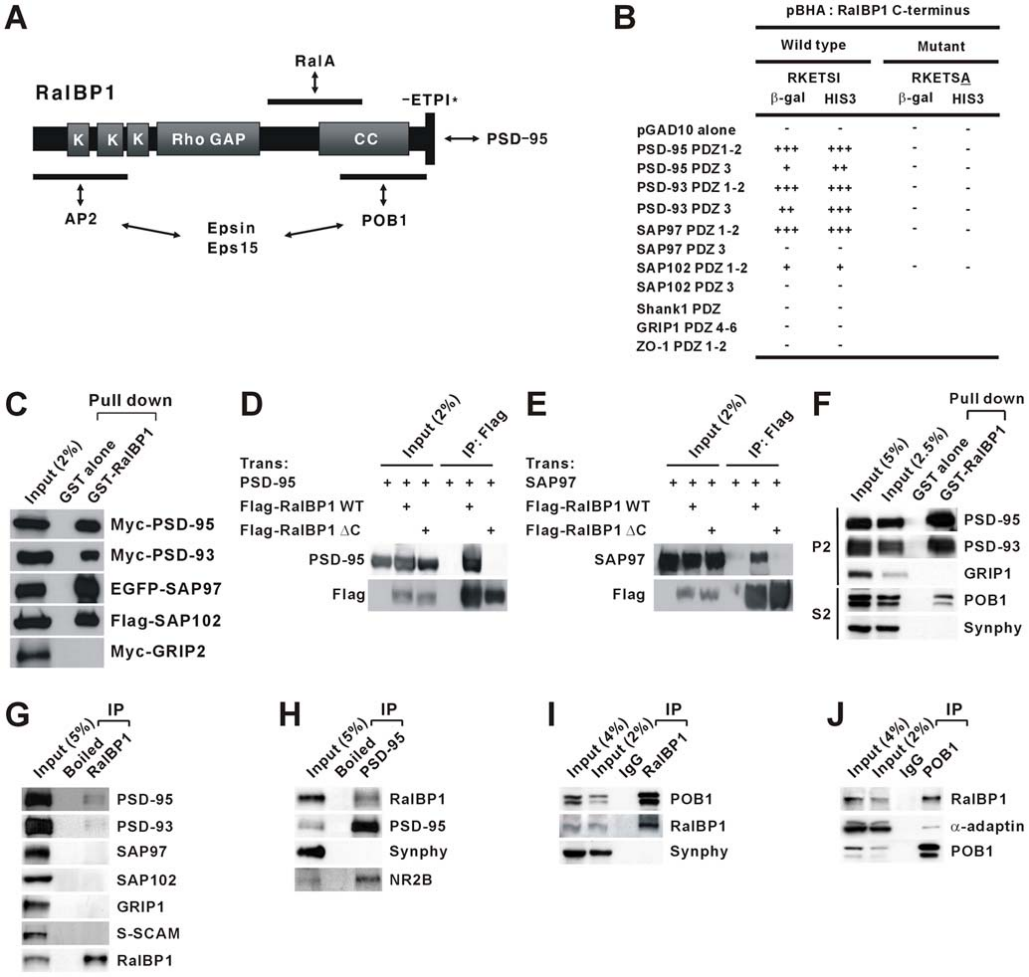
PSD-95 interacts with RalBP1. (A) Domain structure and protein interactions of RalBP1. K, lysine rich region; Rho GAP, Rho GTPase-activating protein domain; CC, coiled coil domain; −ETPI*, the last four residues of rat or mouse RalBP1. Regions of RalBP1 involved in protein interactions are indicated by horizontal lines. Protein interactions are indicated by bidirectional arrows. (B) The RalBP1 C-terminus (aa 410–655; human) interacts with PDZ domains of PSD-95 family proteins (PSD-95, PSD-93/chapsyn-110, SAP97, and SAP102) in yeast two-hybrid assays. Point mutation at the last residue (I655A) of RalBP1 eliminates its PDZ interaction with PSD-95 family proteins. PDZ domains from Shank1, GRIP1, and ZO-1 were used as controls. β-Galactosidase (β-gal) activity: +++, <45 min; ++, 45–90 min; +, 90–240 min; −, no significant β-gal activity. HIS3 activity: +++, >60%; ++, 30%–60%; +, 10%–30%; −, no significant growth. (C) GST fusion proteins of RalBP1 (aa 410–655) pull down PSD-95 family proteins expressed in HEK293T cells. GRIP2, a control PDZ protein; Myc, EGFP, and Flag, epitope tags. (D, E) RalBP1 forms a complex with PSD-95 (D) and SAP97 (E) in HEK293T cells. RalBP1 ΔC, a mutant RalBP1 that lacks the last four residues and, thus, PSD-95 interaction; Trans, Transfection; WT, wild type; IP, immunoprecipitation. (F) GST-RalBP1 pulls down PSD-95, PSD-93, and POB1, but not GRIP1 or synaptophysin (SynPhy; negative controls) from brain extracts. Deoxycholate (1%) extracts of the crude synaptosomal (P2) fraction, or the S2 fraction (supernatant after P2 precipitation), of adult (6 wk) rat brain were pulled down by GST-RalBP1 and immunoblotted with the indicated antibodies. (G, H) RalBP1 coprecipitates with PSD-95 and PSD-93 but does not form a detectable complex with SAP97, SAP102, GRIP1, and S-SCAM in the brain. Deoxycholate (1%) extracts of the crude synaptosomal fraction of adult rat brain were immunoprecipitated and immunoblotted. Boiled, boiled PSD-95 or RalBP1 antibodies; NR2B, NMDA glutamate receptor subunit 2 (a positive control). (I, J) RalBP1 forms a tight complex with POB1 in the brain. The S2 fraction of adult rat brain was immunoprecipitated and immunoblotted. α-adaptin, a subunit of the AP2 adaptor complex known to associate with POB1 (a positive control).

Yeast two-hybrid screens with PSD-95 identified RalBP1 as a novel PSD-95-interacting protein, which is coupled to other endocytic proteins including POB1, RalA, AP2, epsin, and Eps15 (Figure 1A) [34],[36]. The PDZ-binding motif at the RalBP1 C-terminus interacted with the PDZ domains of PSD-95 (Figure 1B). RalBP1 binding to PSD-95 was further confirmed by in vitro and in vivo pull down and coimmunoprecipitation assays (Figure 1C–1H). In addition to interacting with PSD-95, RalBP1 formed an in vivo complex with PSD-93/chapsyn-110 (a PSD-95 relative), but no associations of other PSD-95 family proteins (SAP97 and SAP102) with RalBP1 were detected. RalBP1 also formed a complex with POB1 and α-adaptin (a subunit of the AP2 complex) in the brain (Figure 1I and 1J). Notably, RalBP1 tightly associated with POB1, but relatively weakly with α-adaptin and PSD-95.

### Expression Patterns of RalBP1, RalA, and POB1 in the Brain

In situ hybridization revealed that mRNAs of RalBP1, RalA, and POB1 are widely expressed in various brain regions (Figure S1). The three different mRNAs showed overlapping as well as distinct distribution patterns.

In the brain, RalBP1 and POB1 antibodies recognized a single and double band, respectively (Figure S2A and S2B). RalBP1, RalA, and POB1 proteins were most abundant in the brain, compared to other tissues (Figure S2C). RalBP1, RalA, and POB1 proteins were detected in various brain regions (Figure S2D), consistent with the in situ results. Expression levels of RalBP1 and RalA proteins remained largely unchanged during postnatal brain development, whereas POB1 and α-adaptin showed age-dependent increases (Figure S2E).

In subcellular brain fractions, RalA was mainly detected in crude synaptosomal (P2) and synaptic membrane (LP1) fractions (P21 and 6 wk), similar to PSD-95 (Figure S2F). This is consistent with the previous reports that RalA is present in postsynaptic protein complexes [37],[38]. In contrast, RalBP1 and POB1 were largely found in cytosolic (S3) and microsomal (P3) fractions. RalA was detected in the PSD I fraction and weakly in PSD II and III fractions (Figure S2G), suggesting that RalA is not tightly associated with the PSD, although it is mainly detected in synaptic fractions. RalBP1 was minimally detected in PSD fractions.

### Phosphorylation of RalBP1 at C-terminal Thr 645 Inhibits PSD-95 Binding

**Figure 2:**
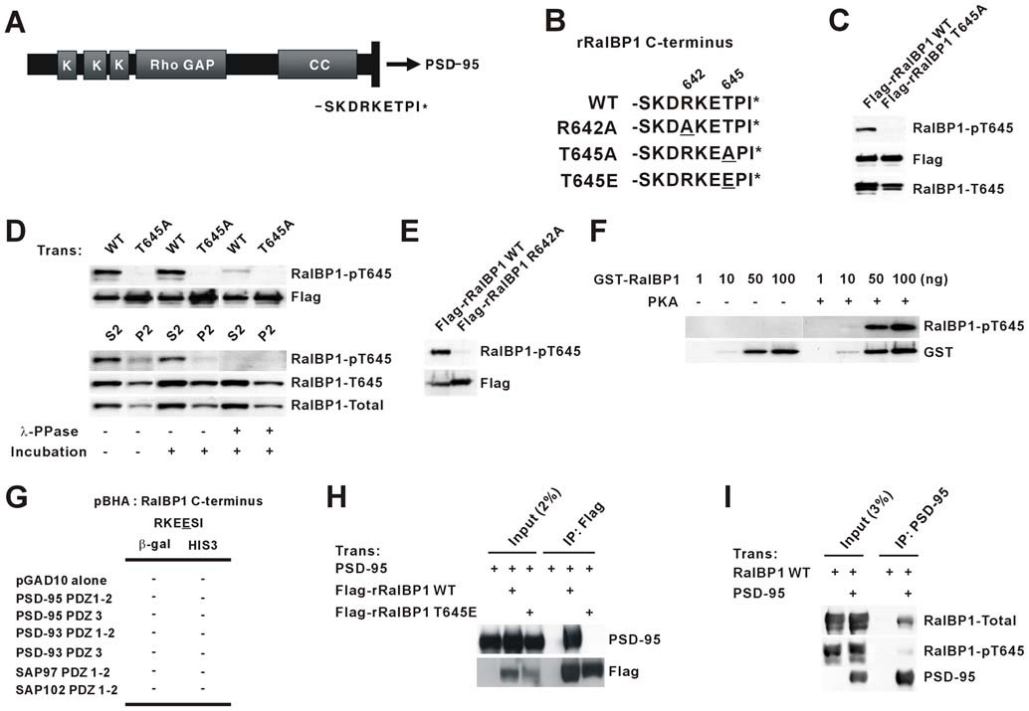
Phosphorylation of RalBP1 at C-terminal Thr 645 inhibits PSD-95 binding. (A) C-terminal aa sequence (SKDRKETPI*) of rat RalBP1 (rRalBP1). (B) Amino acid sequence of WT and mutant rRalBP1 C-termini. (C) RalBP1 is phosphorylated at Thr 645 in heterologous cells. Lysates of HEK293T cells transfected with Flag-rRalBP1 (WT or T645A) were immunoblotted with the indicated antibodies. RalBP1-pT645, phosphopeptide antibody; RalBP1-T645; non-phosphopeptide antibody. Flag antibodies were used for normalization. (D) λ-phosphatase incubation dephosphorylates phospho-Thr 645 from RalBP1 expressed in heterologous cells and in the brain. Flag-rRalBP1 (WT or T645A) expressed in HEK293T cells, or endogenous RalBP1 from rat brain (S2 and P2 fractions), on nitrocellulose membranes was incubated with λ-phosphatase, followed by immunoblotting with RalBP1-pT645, RalBP1-T645, and RalBP1-Total antibodies. RalBP1-Total was raised against the C-terminal third of RalBP1 and thus recognizes both phospho- and non-phospho-RalBP1; λ-PPase, λ-phosphatase. (E) A mutation (R642A; −5 position) at the RalBP1 C-terminus inhibits RalBP1 phosphorylation. Lysates of HEK293T cells transfected with Flag-rRalBP1 (WT or R642A) were immunoblotted with the indicated antibodies. (F) PKA directly phosphorylates RalBP1 on Thr 645 in vitro. Increasing amounts of GST-RalBP1 were incubated with the catalytic subunit of PKA, followed by the immunoblot analysis with GST and RalBP1-pT645 antibodies. (G) RalBP1 T645E, a phosphomimetic mutant, fails to interact with PDZ domains from PSD-95 family proteins (PSD-95, PSD-93, SAP97, and SAP102) in yeast two-hybrid assays. Note that the human RalBP1 C-terminus used in this experiment ends with RKETSI (human) instead of RKETPI (rat; Figure 1A). (H) RalBP1 T645E fails to form a complex with PSD-95 in heterologous cells. Lysates of HEK293T cells doubly transfected with PSD-95+Flag-rRalBP1 (WT or T645E), or singly with PSD-95, were immunoprecipitated with Flag antibodies and immunoblotted for Flag and PSD-95. (I) Phosphorylated RalBP1 shows a reduced association with PSD-95, compared to total (phosphorylated+non-phosphorylated) RalBP1. Lysates of HEK293T cells doubly transfected with PSD-95 and RalBP1 (WT), or singly with RalBP1, were immunoprecipitated with PSD-95 antibodies and immunoblotted.

The limited subcellular overlap between RalBP1 and PSD-95 (Figure S2F) suggests that their interaction might be regulated. Because PDZ interactions can be regulated by phosphorylation [39], we tested if Thr 645 (−2 position) at the RalBP1 C-terminus can be phosphorylated (Figure 2A). To this end, we generated a RalBP1 antibody that selectively recognizes RalBP1 proteins with phosphorylated Thr 645. This antibody could detect wild-type (WT) RalBP1 but not a RalBP1 T645A mutant (Figure 2B and 2C). λ-Phosphatase digestion of RalBP1 proteins expressed in vitro and in vivo substantially weakened protein detection by phospho-specific antibodies (Figure 2D). An upstream mutation in RalBP1 (R642A; −5 position) abolished the phosphorylation (Figure 2E), indicating that R642 is important for RalBP1 phosphorylation. Of note, the RKET sequence in the RalBP1 C-terminus (−5 to −2 positions) matches the consensus sequence for protein kinase A (PKA) phosphorylation. Consistent with this possibility, PKA could directly phosphorylate RalBP1 in vitro (Figure 2F).

A phosphomimetic RalBP1 mutant (T645E) failed to interact with PSD-95 in yeast two-hybrid and in vitro coprecipitation assays (Figure 2G and 2H). RalBP1 proteins phosphorylated at T645 showed reduced biochemical association with PSD-95 in HEK293T cells, compared to total (phosphorylated+non-phosphorylated) RalBP1 (Figure 2I). These results suggest that RalBP1 is phosphorylated at Thr 645 in vivo, and this inhibits PSD-95 binding.

### RalBP1 Is Dephosphorylated by NMDAR Activation via PP1 and Rephosphorylated by PKA

**Figure 3:**
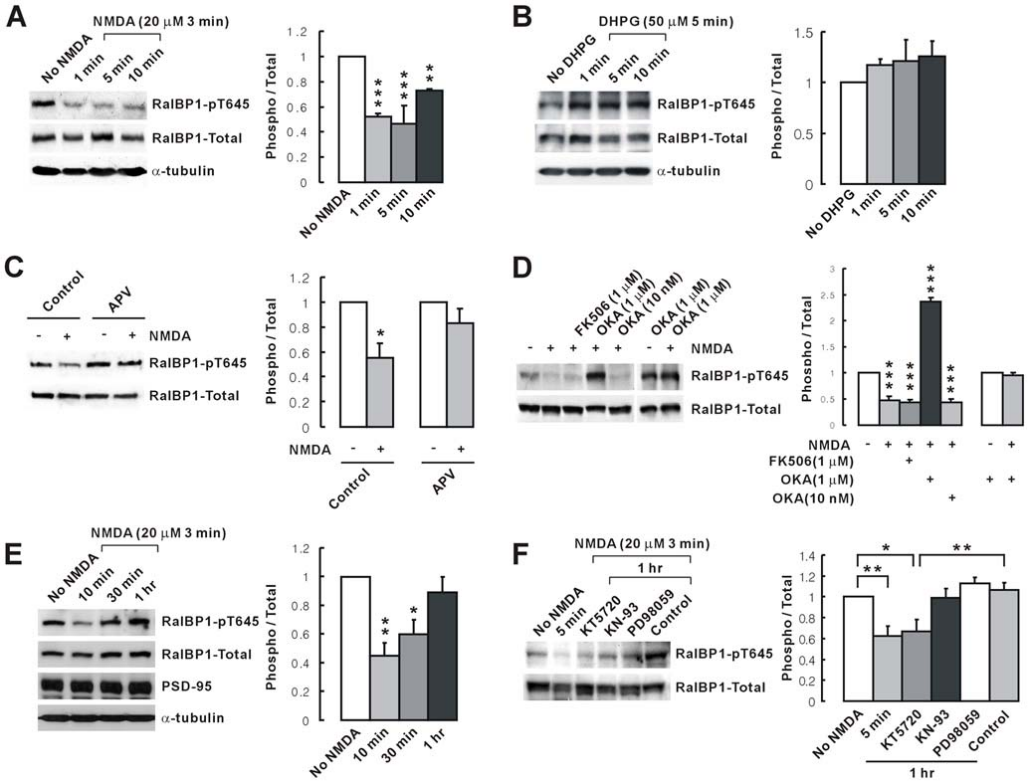
RalBP1 is dephosphorylated by NMDAR activation through PP1 and rephosphorylated by PKA. (A) NMDA treatment rapidly dephosphorylates RalBP1. Cultured hippocampal neurons (>DIV 21) were treated with NMDA (20 µM for 3 min) and incubated in the same media without NMDA for 1, 5, or 10 min, followed by immunoblotting with RalBP1-phospho (pT645), RalBP1-Total, and α-tubulin (normalization) antibodies. The ratio of phosphor/total RalBP1 was normalized to “No NMDA” control. Mean±SEM; *n* = 3, ** *p*<0.01, *** *p*<0.001, ANOVA. (B) mGluR activation by DHPG (50 µM, 5 min) does not affect RalBP1 phosphorylation. (C) NMDAR inhibition by APV 5 min prior to and during NMDA treatment blocks RalBP1 dephosphorylation. *n* = 4, * *p*<0.05. (D) PP1 inhibition 1 h prior to and during NMDA treatment blocks RalBP1 dephosphorylation. OKA, okadaic acid. The increased signal by 1 µM OKA may reflect a reduced basal RalBP1 dephosphorylation. Experiments for the last two lanes were performed independently, and the results were normalized to OKA (1 µM) without NMDA treatment. *n* = 3, *** *p*<0.001, ANOVA. (E) Time course of RalBP1 rephosphorylation. After NMDA washout, neurons were recovered for the indicated amounts of time. *n* = 3, * *p*<0.05, ** *p*<0.01, ANOVA. (F) PKA inhibition during the 1 h recovery blocks RalBP1 rephosphorylation. Five min, 5 min after NMDA; control, no inhibitor. *n* = 3–5, * *p*<0.05, ** *p*<0.01, ANOVA.

We next tested if NMDAR activation, via protein phosphatases, dephosphorylates RalBP1. NMDAR activation by NMDA treatment induces AMPAR endocytosis in cultured neurons [11],[40] and LTD in slices [12]. NMDA treatment (20 µM for 3 min) of cultured hippocampal neurons induced a rapid and significant (∼50%) dephosphorylation of RalBP1 at Thr 645 (Figure 3A). As metabotropic glutamate receptor (mGluR)–dependent LTD also involves AMPAR endocytosis [1], we tested if mGluR activation leads to the dephosphorylation of RalBP1. DHPG, a group I mGluR agonist, however, did not induce RalBP1 dephosphorylation (Figure 3B).

RalBP1 dephosphorylation induced by NMDA treatment was blocked by APV, an NMDAR antagonist (Figure 3C). RalBP1 dephosphorylation was blocked by okadaic acid (1 µM), which inhibits both protein phosphatase 1 and 2A (PP1 and PP2A) (Figure 3D). In contrast, RalBP1 dephosphorylation was not affected by low-concentration okadaic acid (10 nM), which inhibits only PP2A, or FK506, an inhibitor of PP2B (calcineurin), indicating that PP1 is important. Notably, okadaic acid (1 µM) increased RalBP1 phosphorylation before NMDA treatment (Figure 3D), indicating that PP1 also mediates basal dephosphorylation of RalBP1.

After NMDA washout, RalBP1 was rephosphorylated to near-normal levels in ∼1 h (Figure 3E). This rephosphorylation was blocked by KT5720 (1 µM), a PKA inhibitor, but not by KN-93 (10 µM) and PD98059 (25 µM), which inhibit CaM kinases [41] and MEK, respectively (Figure 3F). These results suggest that RalBP1 is rapidly dephosphorylated by NMDAR activation and relatively slowly rephosphorylated by PKA.

### NMDA Treatment Induces Activation of RalA, and Activated RalA Binds and Translocates RalBP1 to Dendritic Spines

**Figure 4:**
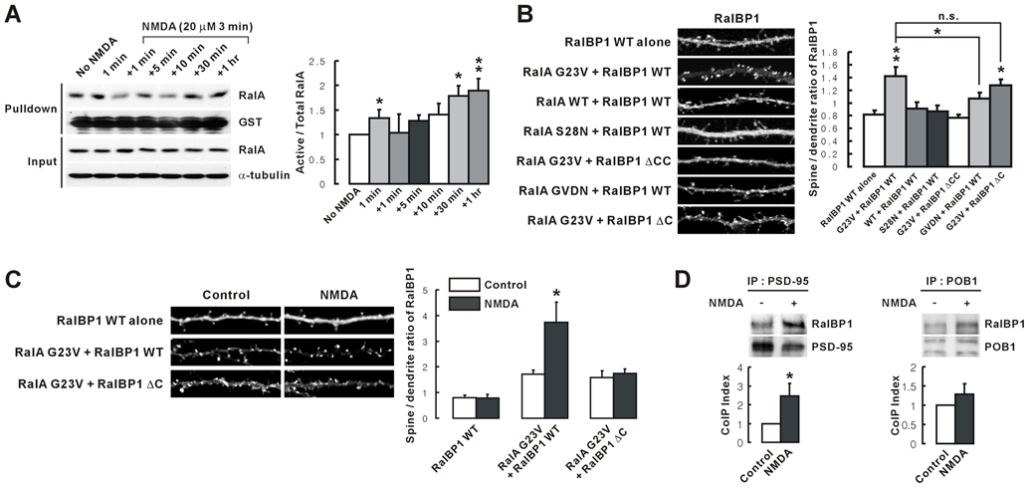
NMDAR activation induces RalA activation, and activated RalA and PSD-95 act together to bind and translocate dephosphorylated RalBP1 to spines. (A) NMDA treatment rapidly induces RalA activation. Neurons (>DIV 21) were incubated with NMDA for 1 min, or for 3 min+indicated amount of time without NMDA, followed by pull down of active RalA by GST-RalBD and immunoblotting. The ratio of active/total RalA was normalized to control (No NMDA). *n* = 5–8, * *p*<0.05, ** *p*<0.01, ANOVA. (B) Active RalA (RalA G23V) binds and translocates RalBP1 to dendritic spines. Neurons cotransfected with RalA (WT and mutants) and RalBP1 constructs (WT and mutants; DIV 17–18) were immunostained for RalBP1, followed by the analysis of spine localization (spine/dendrite ratio) of RalBP1. *n* = 6–13, * *p*<0.05, ** *p*<0.01; ANOVA. n.s., not significant. (C) NMDA treatment further enhances RalA-dependent spine translocation of RalBP1. Neurons cotransfected with RalBP1 alone, or RalA G23V+RalBP1 (WT or ΔC) (DIV 17–18), were treated with NMDA (3 min), followed by staining for RalBP1. *n* = 12–18, * *p*<0.05. (D) NMDA treatment increases coprecipitation between RalBP1 and PSD-95, but not that between RalBP1 and POB1 (control). Lysates of NMDA-treated neurons (>DIV 18) were immunoprecipitated with PSD-95, or POB1, antibodies and immunoblotted. *n* = 4–5, * *p*<0.05.

Because RalA activation is regulated by Ras, Rap, and calcium/calmodulin [29],[30], which act downstream of NMDAR activation [1],[42], we tested if NMDAR activation leads to the activation of RalA. NMDA treatment of cultured neurons induced RalA activation, measured by pull down assays (Figure 4A). The RalA activation consisted of two phases; a rapid (<1 min) and small increase followed by a slow and bigger increase.

In cultured neurons, RalBP1 expressed alone showed a widespread distribution pattern in dendrites (Figure 4B). Interestingly, coexpression of a constitutively active form of RalA (G23V) with RalBP1 induced a marked translocation of RalBP1 to dendritic spines, whereas WT and dominant negative RalA (S28N; constitutively in the GDP-bound state) had no effect (Figure 4B). RalBP1-enriched spines were positive for PSD-95 (Figure S3), indicating that RalBP1 was translocated to synaptic sites. RalBP1 ΔCC, which lacks the CC domain that is involved in RalA binding, showed no significant RalA-dependent spine translocation, indicating that the direct binding of RalBP1 to RalA is important. A RalA G23V mutant with weakened RalBP1 binding (G23VD49N; termed GVDN) induced a spine translocation of RalBP1 that is smaller than that of RalBP1 coexpressed with RalA G23V, further suggesting that RalA directly recruits RalBP1 to spines. Notably, RalBP1 ΔC, which lacks PSD-95 binding, showed a RalA-dependent spine translocation similar to that of WT RalBP1, indicating that activated RalA alone is sufficient to induce spine translocation of RalBP1. These results suggest that NMDAR activation induces RalA activation and that activated RalA binds and translocates RalBP1 to dendritic spines.

### RalBP1 Dephosphorylation Combined with RalA Activation Further Promotes Spine Translocation of RalBP1

We next tested whether RalBP1 dephosphorylation induced by NMDAR activation affects RalA-induced spine translocation of RalBP1. NMDA treatment of neurons coexpressing RalA G23V and RalBP1 further increased RalA-dependent spine translocation of RalBP1 (Figure 4C). In contrast, such increases were not observed in control neurons expressing RalA G23V and RalBP1 ΔC, indicating that NMDA-induced RalBP1 binding to PSD-95 is important. RalBP1 that was transfected alone was not translocated to spines upon NMDA treatment, indicating that dephosphorylation of RalBP1 alone is not sufficient to induce RalBP1 translocation to spines. Spine morphology, as measured by spine head area, was not changed by overexpression of RalA and RalBP1 constructs (WT and mutants), or by NMDA treatment of the transfected neurons (Figure S4). These results suggest that binding of dephosphorylated RalBP1 to PSD-95, combined with RalBP1 binding to activated RalA, further promotes synaptic localization of RalBP1.

Biochemically, NMDA treatment of cultured neurons significantly increased coimmunoprecipitation of RalBP1 and PSD-95, but not of RalBP1 and POB1 (Figure 4D). Whether the association between RalBP1 and RalA is affected by NMDA treatment could not be determined because RalBP1 did not coprecipitate with RalA under our experimental conditions, possibly owing to the transient nature of RalA binding to RalBP1. In support of this interpretation, RalBP1 did not form a complex with RalA WT, in contrast to the strong association of RalBP1 with RalA G23V (Figure S5A).

The results described thus far indicate that a ternary complex containing RalA, RalBP1, and PSD-95 might be formed in a regulated manner. Because the formation of a RalA-RalBP1 complex could not be demonstrated in vivo, we tested this possibility in heterologous cells using RalA G23V. In HEK293T cells, RalA G23V formed a ternary complex with RalBP1 and PSD-95 (Figure S5B). In addition, RalA, which is mainly associated with the plasma membrane by geranyl-geranylation, induced translocation of PSD-95 to the plasma membrane in HEK293T cells coexpressing RalBP1 WT, but not in those coexpressing RalBP1 ΔC that lacks PSD-95 binding ability (Figure S5C). Collectively, these results indicate that RalA and PSD-95 act together to bind and translocate RalBP1 to synapses.

### NMDAR Activation by Low-Frequency Electrical Stimulation (LFS) Induces RalBP1 Dephosphorylation

**Figure 5:**
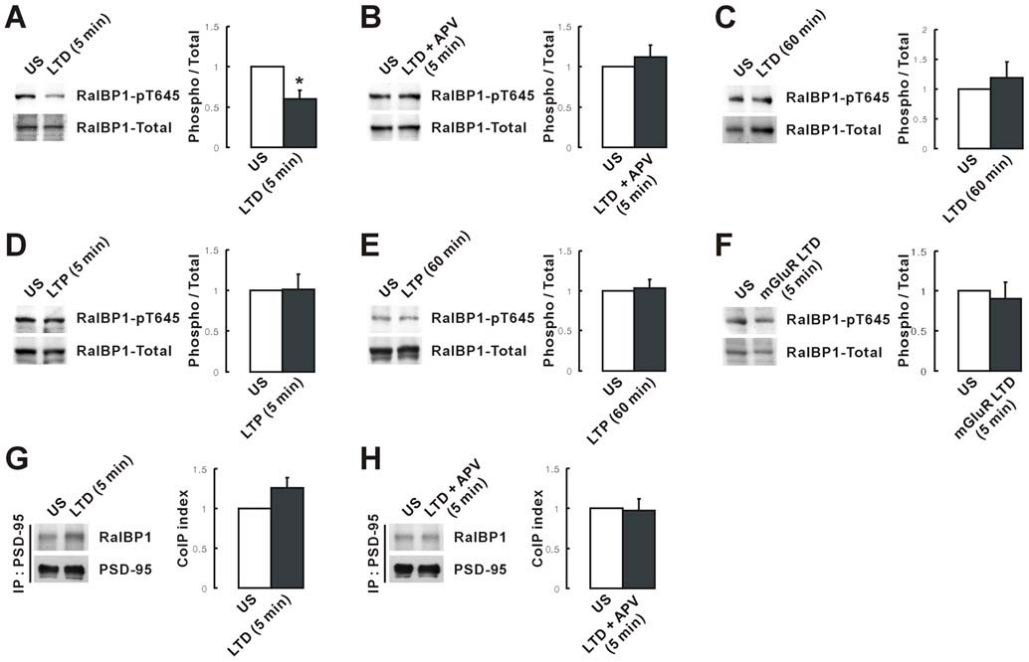
NMDAR activation by low-frequency electrical stimulation induces RalBP1 dephosphorylation. (A–C) Electrical low-frequency stimulation (LFS; 1 Hz, 900 pulses) of the CA1 region of acute hippocampal slices induces RalBP1 dephosphorylation in 5 min (A), which is blocked by APV (NMDAR antagonist; B) and is returned to normal ranges in 60 min after the stimulation (C). *n* = 6, * *p*<0.05, Student's *t*-test. (D–F) RalBP1 phosphorylation is unaffected by LTP induced by TBS (5 min and 60 min; D, E) and mGluR LTD induced by PP-LFS combined with APV (5 min; F). (G, H) LFS does not enhance the association between RalBP1 and PSD-95. LFS-stimulated CA1 region of hippocampal slices were detergent extracted and immunoprecipitated with PSD-95 antibodies followed by immunoblot analysis with PSD-95 and RalBP1 antibodies.

The results described thus far are based on experiments using NMDA treatment to induce RalA activation and RalBP1 dephosphorylation. Bath application of NMDA leads to activation of both synaptic and extrasynaptic NMDARs, which can be coupled to different signal transduction pathways [43],[44]; therefore, we attempted NMDAR activation by LFS (1 Hz, 900 pulses), which likely enhances activation of synaptic NMDARs [45]. The levels of RalBP1 phosphorylation at T645 were significantly decreased by LFS given to the CA1 region of hippocampal slices, an effect that was blocked by the NMDAR antagonist APV (Figure 5A and 5B). RalBP1 phosphorylation levels returned to a normal range 60 min after LFS (Figure 5C), a result similar to that obtained in NMDA-treated cultured neurons. In contrast, neither induction of LTP by theta-burst stimulation (TBS) nor induction of LTD by paired-pulse LFS (PP-LFS, 50 ms interstimulus interval) in the presence of APV induced RalBP1 dephosphorylation (Figure 5D–5F). Although there was a tendency for LFS to increase coimmunoprecipitation of RalBP1 and PSD-95 compared with that of LFS and APV (Figure 5G–5H), this difference did not reach statistical significance (*p* = 0.1; *n* = 6). This result is in contrast to the enhanced coprecipitation of RalBP1 and PSD-95 observed in NMDA-treated cultured neurons (Figure 4D). This discrepancy might be attributable to the fact that PSD-95 proteins in slices are more difficult to extract than those in cultured neurons, leading to a decrease in the efficiency of coprecipitation between PSD-95 and RalBP1.

### RalBP1 and RalA Are Required for NMDA-induced AMPAR Endocytosis and LTD

RalBP1, an endocytic adaptor, translocated to synapses by NMDAR activation might regulate AMPAR endocytosis. To test this hypothesis, we attempted knockdown of RalBP1 and RalA by shRNA constructs, which reduced expression of exogenous RalBP1 and RalA by 78% and 86%, respectively, in HEK293T cells, and by 90% and 77%, respectively, in cultured neurons (Figure S6). Knockdown of endogenous proteins could not be observed due to the lack of suitable antibodies.

**Figure 6:**
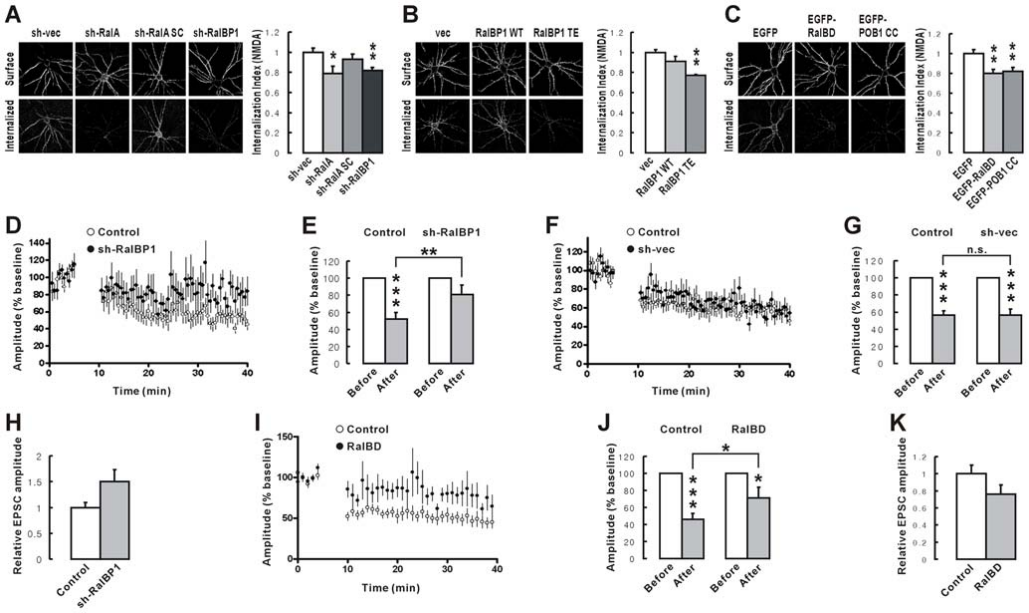
RalBP1 and RalA are required for NMDA-induced AMPAR endocytosis and LTD. (A) Knockdown of RalBP1 and RalA suppresses NMDA-induced endocytosis of GluR2. Cultured neurons transfected with HA-GluR2+sh-(RalBP1, RalA, SC/scrambled, or vec/empty vector) (DIV 16–20) were subject to the antibody feeding assay under NMDA treatment condition (20 µM, 3 min). The internalization index (the ratio of internalized to internalized+surface receptors) was normalized to sh-vec control. *n* = 14–21, * *p*<0.05, ** *p*<0.01, Student's *t*-test. (B) Overexpression of phosphomimetic RalBP1 TE (T645E), but not WT RalBP1, suppresses NMDA-induced GluR2 endocytosis. vec, vector alone. *n* = 17–22, ** *p*<0.01. (C) Overexpression of the RalBD and the POB1 CC, which inhibits RalA (active) and RalBP1, respectively, reduces NMDA-induced GluR2 endocytosis. *n* = 15–27, ** *p*<0.01. (D–G) RalBP1 knockdown suppresses paring-induced LTD at SC-CA1 synapses. CA1 pyramidal neurons in slice culture were transfected with sh-RalBP1 (D), or sh-vec (F) (DIV 3/4–6/7), followed by LTD induction by paring 300 pulses at 1 Hz at −45 mV. Average AMPAR EPSCs 25–30 min after LFS were measured from pairs of shRNA-expressing and untransfected neurons. Histograms (E and G) indicate EPSC amplitudes after LTD induction normalized to those before the induction (baseline). *n* = 10, *** *p*<0.001. The significance between the two LTD values was determined by Student's paired *t*-test. ** *p*<0.01, n.s., not significant. (H) Normal synaptic transmission in sh-RalBP1-expressing CA1 pyramidal neurons. EPSC amplitudes were normalized to untransfected control neurons in the same pair. (I–J) Overexpression of RalBD in CA1 pyramidal neurons reduces LTD at SC-CA1 synapses. *n* = 9, * *p*<0.05, *** *p*<0.001. The significance between the two LTD values was determined by Student's paired *t*-test. * *p*<0.05. (K) Normal synaptic transmission in RalBD-expressing neurons.

In cultured neurons, knockdown of RalBP1 and RalA significantly reduced NMDA-induced endocytosis of the GluR2 subunit of AMPARs (Figure 6A). A scrambled version of RalA shRNA had no effect. shRNA-resistant expression constructs of RalBP1 and RalA coexpressed with RalBP1 and RalA shRNAs, respectively, rescued the knockdown effects (Figure S7). Overexpression of RalBP1 TE, a phosphomimetic RalBP1 mutant that lacks PSD-95 binding, inhibited NMDA-induced GluR2 endocytosis, while WT RalBP1 did not (Figure 6B), suggesting that RalBP1 binding to PSD-95 is important. In addition, the CC domain of POB1 (POB1 CC), which binds and inhibits RalBP1, significantly reduced NMDA-induced GluR2 endocytosis (Figure 6C).

Further supporting the importance of RalA, the Ral binding domain of RalBP1 (RalBD), which binds and inhibits only active RalA, significantly reduced NMDA-induced GluR2 endocytosis (Figure 6C). Furthermore, RalA S28N (dominant negative) and RalA GVDN (a RalA G23V mutant with weakened RalBP1 binding) inhibited NMDA-induced GluR2 endocytosis, whereas RalA WT and RalA G23V had no effect (Figure S8A). These results suggest that RalBP1 and RalA are required for NMDA-induced endocytosis of GluR2.

In hippocampal slice culture, RalBP1 knockdown in CA1 pyramidal neurons significantly reduced paring-induced LTD at Schaffer collateral (SC)–CA1 pyramidal cell (CA1) synapses (∼81% of baseline; *p* = 0.12 compared to before paring; Student's unpaired *t*-test), relative to untransfected control neurons (∼52% of baseline; *** *p*<0.001) (Figure 6D and 6E). The LTD magnitudes from neurons expressing RalBP1 shRNA and untransfected control neurons (∼81% and ∼52% of baseline, respectively) were significantly different (** *p*<0.01, Student's paired *t*-test). In contrast, neurons transfected with empty shRNA vector showed an LTD magnitude comparable to that of untransfected neurons (Figure 6F and 6G). RalBP1 knockdown did not affect basal synaptic transmission, as measured by amplitudes of evoked excitatory postsynaptic currents (EPSCs) (Figure 6H).

Supporting the role of RalA in LTD regulation, the RalA-inhibiting construct RalBD significantly reduced paring-induced LTD (∼71% of baseline; * *p*<0.05 compared to before paring; Student's unpaired *t*-test), relative to untransfected control neurons (∼46% of baseline; *** *p*<0.001). LTD magnitudes observed in RalBD overexpressing and untransfected neurons were significantly different (* *p*<0.05; Student's paired *t*-test; Figure 6I and 6J). Basal transmission was unaffected by RalBD overexpression (Figure 6K). These results suggest that RalBP1 and RalA are required for LTD induction.

### RalA, but not RalBP1, Inhibits Basal AMPAR Endocytosis in a GTP-independent Manner

**Figure 7:**
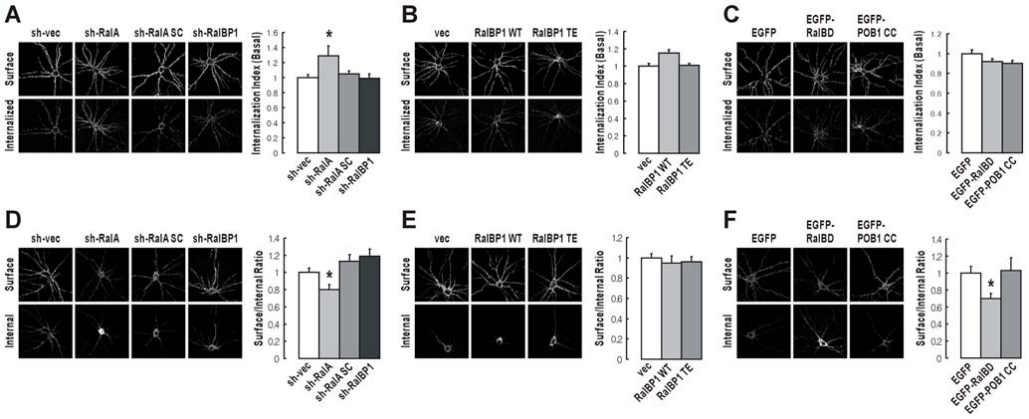
RalA, but not RalBP1, inhibits basal AMPAR endocytosis in a GTP-independent manner and is required for the maintenance of surface AMPARs. (A) Knockdown of RalA, but not RalBP1, increases GluR2 endocytosis under basal conditions (no NMDA treatment). Cultured neurons doubly transfected with HA-GluR2 and the indicated shRNA constructs (DIV 16–20) were subject to the antibody feeding assay in the absence of NMDA treatment. The internalization index was normalized to sh-vec control. *n* = 18–20, * *p*<0.05, Student's *t*-test. (B) Overexpression of RalBP1 WT or RalBP1 TE does not affect basal endocytosis of GluR2. Cultured neurons transfected with HA-GluR2 and RalBP1 (WT, TE, or vector alone) were subject to the antibody feeding assay. The internalization index was normalized to vector alone control. *n* = 23–26. (C) Dominant negative inhibition of RalA and RalBP1 by RalBD and POB1 CC, respectively, has no effect on basal GluR2 endocytosis. Cultured neurons transfected with HA-GluR2 and EGFP-RalBD, or EGFP-POB1 CC, were subject to the antibody feeding assay. The internalization index was normalized to EGFP control. *n* = 16–24. (D) Knockdown of RalA, but not RalBP1, reduces surface expression levels of GluR2. Cultured neurons doubly transfected with HA-GluR2 and the indicated shRNA constructs (DIV 16–20) were measured of the steady-state surface-to-internal ratio of GluR2. *n* = 17–28, * *p*<0.05, Student's *t*-test. (E) Overexpression of RalBP1 WT or RalBP1 TE has no effect on surface AMPAR levels. Cultured neurons doubly transfected with HA-GluR2 and RalBP1 (WT, TE, or vector alone; DIV 16–20) were measured of their surface GluR2 expression. *n* = 13–25. (F) Dominant negative inhibition of active RalA by RalBD, but not the inhibition of RalBP1 by POB1 CC, reduces surface GluR2 levels. Cultured neurons doubly transfected with HA-GluR2 and EGFP-RalBD, EGFP-POB1 CC, or EGFP alone (DIV 16–20) were measured of their surface GluR2 expression. *n* = 15–24, * *p*<0.05, Student's *t*-test.

We next tested whether RalA and RalBP1 regulate AMPAR endocytosis under basal conditions. Intriguingly, basal GluR2 endocytosis in the absence of NMDAR activation was enhanced by the knockdown of RalA, but not RalBP1 (Figure 7A), suggesting that RalA, but not RalBP1, inhibits GluR2 endocytosis under basal conditions. Inhibition of RalBP1 by overexpression of RalBP1 TE and POB1 CC had no effect on basal GluR2 endocytosis (Figure 7B and 7C), further suggesting that RalBP1 does not regulate basal GluR2 endocytosis. Intriguingly, basal GluR2 endocytosis was not affected by overexpression of RalBD (Figure 7C), RalA S28N, or RalA GVDN (Figure S8B). RalBD, RalA S28N, and RalA GVDN commonly interfere with GTP-dependent actions of RalA by trapping activated RalA, blocking RalA activation, and suppressing the binding of activated RalA to RalBP1, respectively. Considering that RalA knockdown reduces total RalA (both active and inactive), these results suggest that RalA inhibits basal AMPAR endocytosis in a GTP-independent (or RalA activation-independent) manner.

### RalA, but not RalBP1, Is Required for the Maintenance of Surface AMPAR Levels

RalA inhibits basal AMPAR endocytosis, so we reasoned that RalA might regulate surface AMPAR levels. Indeed, knockdown of RalA, but not RalBP1, significantly reduced surface levels of GluR2 (Figure 7D), suggesting that surface GluR2 levels are maintained by RalA but not RalBP1. Consistent with this, inhibition of RalBP1 by RalBP1 TE or POB1 CC had no effect on surface GluR2 levels (Figure 7E and 7F).

Interestingly, surface GluR2 levels were reduced by overexpression of RalBD (Figure 7F) or RalA S28N (Figure S8C), which inhibits active RalA, indicating that active RalA is important for the maintenance of surface GluR2 levels. Collectively, these results indicate that both active and inactive RalA are involved in maintaining surface GluR2 levels. Inactive RalA may help maintain surface GluR2 levels by inhibiting basal GluR2 endocytosis (Figures 7A, 7C, and S8B). How then might active RalA contribute to the maintenance of surface GluR2 levels? One possibility is that active RalA might help internalized GluR2 recycle back to the plasma membrane. However, inhibition of active RalA by overexpression of RalA S28N had no effect on GluR2 recycling (Figure S9A). In addition, knockdown of RalA, which reduces total (active+inactive) RalA levels, did not affect GluR2 recycling (Figure S9B), indicating that neither active nor inactive RalA regulate GluR2 recycling. An alternative possibility is that active RalA might regulate synaptic delivery of GluR2 from a cytoplasmic, non-recycling pool, perhaps via the interaction of RalA with the exocyst complex. In support of this possibility, two components of the exocyst complex (Sec8 and Exo70), which interact with active RalA, have been shown to promote surface insertion and synaptic targeting of AMPARs [46].

### Constitutive RalA Activation Combined with RalBP1 Binding to PSD-95 Reduces Surface AMPAR Levels and Occludes NMDA-Induced AMPAR Endocytosis

**Figure 8:**
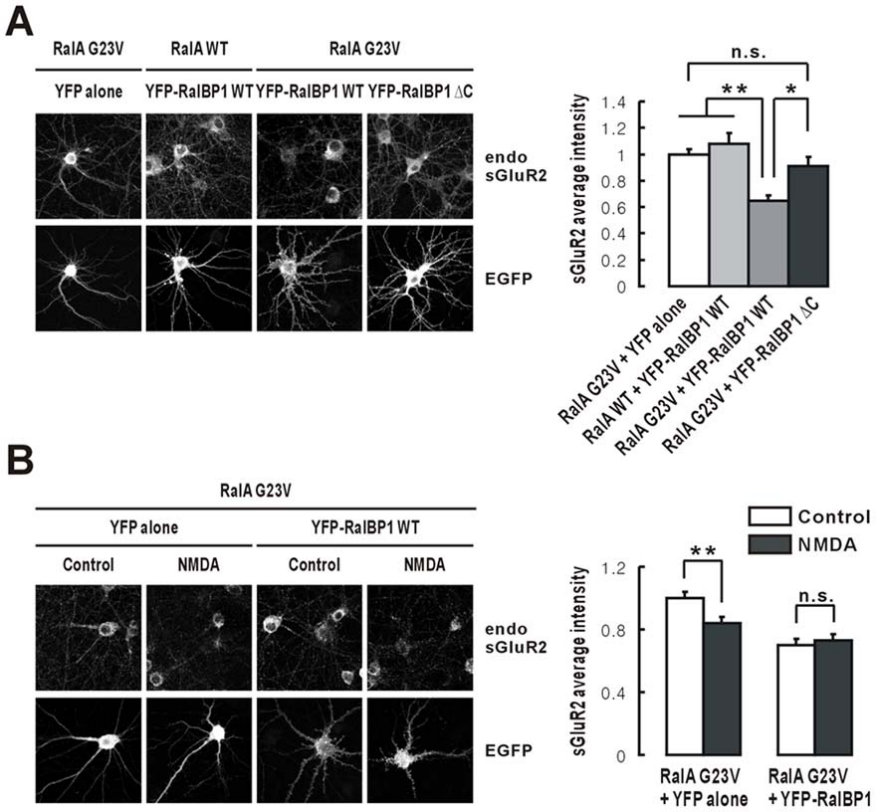
Constitutive RalA activation combined with RalBP1 binding to PSD-95 reduces surface AMPAR levels and occludes NMDA-induced AMPAR endocytosis. (A) Constitutive RalA activation combined with RalBP1 binding to PSD-95 reduces surface levels of AMPARs (GluR2-containing endogenous receptors). Cultured neurons expressing RalA (G23V or WT)+YFP-RalBP1 (WT, ΔC, or YFP alone)+PSD-95 (C-terminally FRB tagged) (DIV 18–20) were stained for surface GluR2 (endogenous), EGFP (for YFP-RalBP1), and PSD-95 (unpublished data). PSD-95 was cotransfected to ensure that the amount of PSD-95 does not become a limiting factor for RalBP1 synaptically translocated by RalA G23V to interact with PSD-95. *n* = 10–16, * *p*<0.05, ** *p*<0.01, ANOVA. n.s., not significant. (B) Constitutive RalA activation combined with RalBP1 binding to PSD-95 occludes NMDA-induced reduction in the levels of surface AMPARs. Neurons expressing RalA G23V+YFP-RalBP1 (or YFP alone)+PSD-95-FRB (DIV 18–20) were treated with NMDA (20 µM, 3 min), followed by 10 min incubation in the absence of NMDA and staining for surface GluR2 (endogenous), EGFP (for YFP-RalBP1), and PSD-95 (unpublished data). *n* = 10–13, ** *p*<0.01.

The results described thus far suggest that two molecular mechanisms, RalA activation and RalBP1 binding to PSD-95, are important for NMDAR-dependent AMPAR endocytosis. We next reasoned that these two mechanisms might be sufficient to induce AMPAR endocytosis in the absence of NMDAR activation. To this end, we transfected cultured neurons with constitutively active RalA (G23V) and RalBP1 (YFP-tagged) and monitored surface levels of endogenous AMPARs, using surface GluR2 antibodies. Intriguingly, surface AMPAR levels in these neurons were significantly reduced in the absence of NMDA treatment, compared to those expressing RalA G23V alone (without RalBP1 coexpression) or those coexpressing WT RalA (not G23V) and RalBP1 (Figure 8A). In contrast, coexpression of RalA G23V and a mutant RalBP1 (ΔC) that lacks PSD-95 binding did not induce a reduction in surface AMPAR levels, relative to the coexpression of RalA G23V and WT RalBP1. These results suggest that RalA activation combined with RalBP1 binding to PSD-95 are sufficient to induce a reduction in surface AMPAR levels in the absence of NMDAR activation, likely through a constitutive endocytosis of AMPARs.

The results described above (Figure 8A) also suggest that a fraction of exogenously expressed RalBP1 proteins is basally dephosphorylated (in the absence of NMDAR activation), and the amount of dephosphorylated RalBP1 proteins is sufficient to bind to both RalA G23V and PSD-95 and induce significant AMPAR endocytosis. In support of this possibility, NMDA treatment of the neurons coexpressing RalA G23V and RalBP1 did not induce AMPAR endocytosis (Figure 8B), suggesting NMDA-induced AMPAR endocytosis was occluded. In contrast, neurons coexpressing RalA G23V alone (without RalBP1 coexpression) showed an NMDA-induced reduction in surface AMPAR levels. It is conceivable that the amount of endogenous RalBP1 proteins, although a fraction of them is dephosphorylated, may not be sufficient to induce AMPAR endocytosis, unless a significant fraction of them is dephosphorylated by NMDAR activation.

### Generation and Characterization of RalBP1−/− Mice

**Figure 9:**
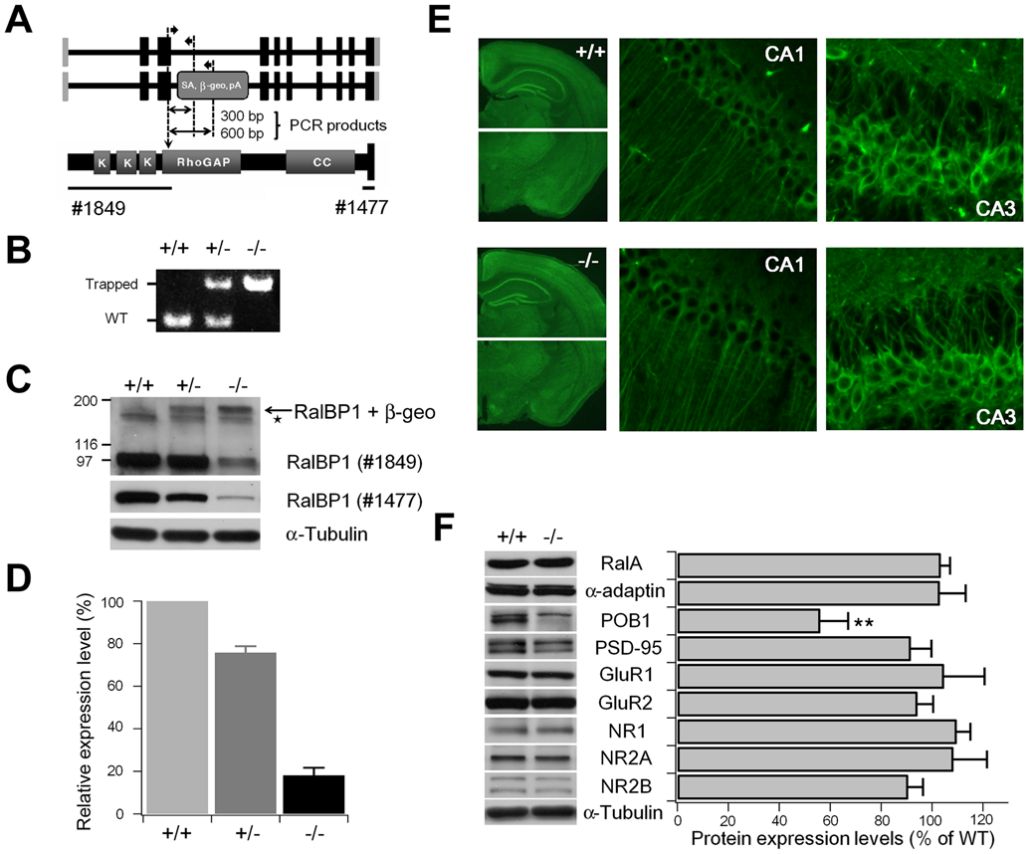
Generation and characterization of RalBP1−/− mice. (A) (top) WT and gene-trapped RalBP1 alleles. Gray and black vertical bars, non-coding and coding regions, respectively; SA, splice acceptor; β-geo, β-galactosidase+neomycin phosphotransferase; PA, polyA. PCR primers and amplified DNA regions are depicted as thick and thin arrows, respectively. (bottom) Domain structure of RalBP1. The site of truncation (arrow; downward and dotted) and antibody regions (lines) are indicated. (B) PCR genotyping of WT and trapped RalBP1 alleles, which produce ∼300 and ∼600 bp fragments, respectively. (C) Reduced RalBP1 expression in RalBP1−/− brain. Brain proteins (S1 fraction) were immunoblotted with two different RalBP1 antibodies (#1849 and #1477; see the panel A for details). Note that there are residual RalBP1 expressions in RalBP1−/− mice, and generation of RalBP1-β-geo fusion proteins. (D) Quantification of the results in (C). (E) Normal brain and neuronal morphologies in RalBP1−/− mice, revealed by immunostaining for NeuN (left; a neuron-specific marker) and MAP2 (middle and right; hippocampus), respectively. (F) Reduced POB1 expression in RalBP1−/− brain. ** *p*<0.01, Student's *t*-test.

To investigate the role of RalBP1 in AMPAR endocytosis and LTD in vivo, we generated RalBP1−/− mice using an ES cell line gene-trapped in the intron between exons 3 and 4 of the RalBP1 gene (Figure 9A). PCR genotyping was used to identify WT and gene-trapped RalBP1 alleles (Figure 9B). Expression levels of RalBP1 proteins in RalBP1−/− brain was ∼18.1%±3.1% (*n* = 8) of WT mice (Figure 9C and 9D), likely due to incomplete gene trapping. The gene trapping generated a small amount of fusion proteins containing RalBP1 (first 235 aa) and β-geo (Figure 9C), which were detected in various brain regions including hippocampus (Figure S10).

No abnormalities were observed in gross morphology of RalBP1−/− brain or in the cellular architecture of RalBP1−/− neurons, as determined by staining for NeuN and MAP2, respectively (Figure 9E). There were no changes in expression levels of RalBP1-interacting proteins such as RalA, α-adaptin, and PSD-95, as well as subunits of AMPARs and NMDARs in RalBP1−/− brain (Figure 9F). Interestingly, however, POB1 expression was significantly decreased by 43.8%±10.9% (*n* = 8), suggesting that RalBP1 is important for the stability of POB1.

### Selective Impairment of NMDAR-Dependent LTD at RalBP1−/− CA1 Synapses

**Figure 10:**
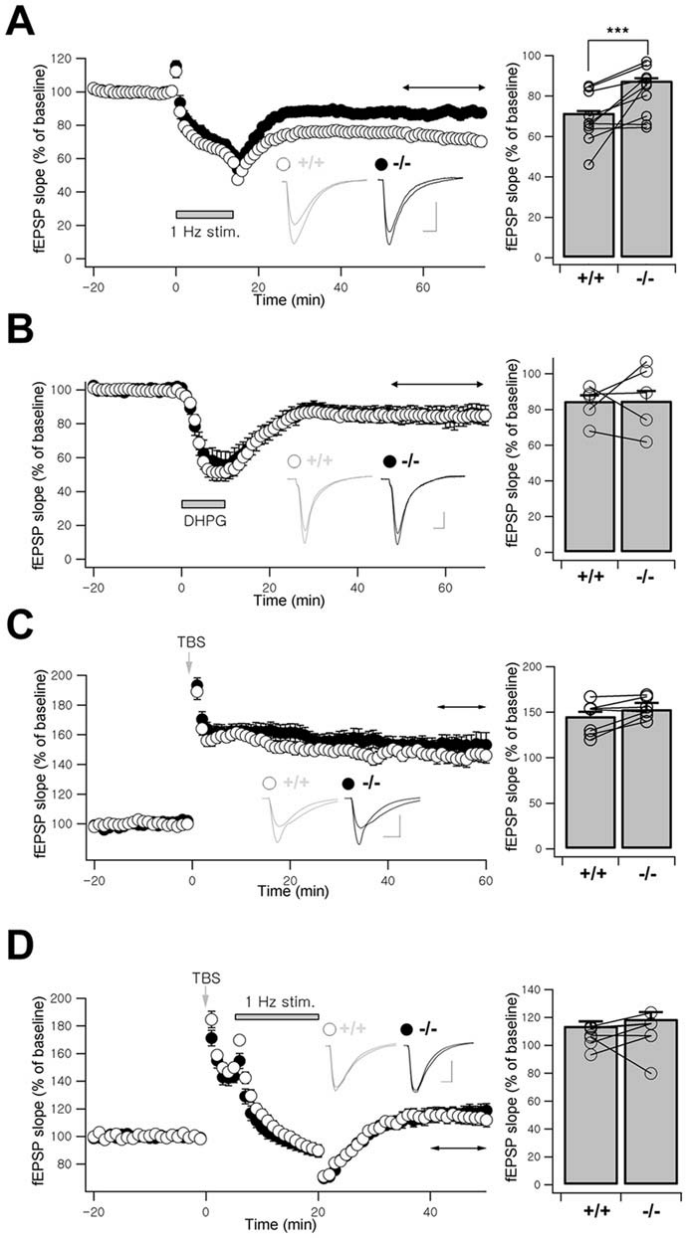
Impaired NMDAR-dependent LTD at RalBP1−/− SC-CA1 pyramidal synapses. (A) LTD induced by LFS (1 Hz, 900 pulses) is significantly attenuated at RalBP1−/− SC-CA1 synapses. Inset indicates fEPSP responses during baseline and 40–60 min after stimulation (horizontal and bidirectional arrow). The bar graph represents mean LTD magnitude (40–60 min, *** *p*<0.001, Student's *t*-test). Connected circles indicate littermate pairs. (B) Similar DHPG-induced LTD at WT (84.67%±3.15%, *n* = 16, 5 animals) and RalBP1−/− SC-CA1 synapses (84.98%±5.36%, *n* = 10, 5). (C) Normal TBS-induced LTP at RalBP1−/− SC-CA1 synapses (WT, 145.66%±4.66%, *n* = 18, 10; RalBP1−/−, 153.31%±6.71%, *n* = 18, 10). (D) Depotentiation induced by LFS after TBS was indistinguishable between genotypes (WT, 113.97%±3.15%, *n* = 11, 7; RalBP1−/−, 118.94%±4.90%, *n* = 12, 7). Scale bars, 5 ms and 0.5 mV (A–D).

We investigated synaptic plasticity at RalBP1−/− hippocampal SC-CA1 synapses. LFS for LTD induction (1 Hz, 900 stimulations) induced robust synaptic depression in WT slices (17–21 d) that averaged 71.6%±0.9% (*n* = 25 slices, 10 animals) (Figure 10A). In contrast, LTD induction in RalBP1−/− mice was significantly attenuated (87.6%±1.1%; *n* = 24, 10 animals; *** *p*<0.001), despite that ∼18% of RalBP1 proteins are still expressed. Inhibition of NMDAR by APV during LFS abolished the difference between the two genotypes (unpublished data).

Hippocampal SC-CA1 synapses also exhibited mGluR-dependent LTD, which does not require protein phosphatase [1]. Bath application of DHPG (mGluR agonist) induced stable depression in both WT and RalBP1−/− slices (8 wk), with magnitude of depression essentially identical throughout the recording (Figure 10B). These results suggest that the reduced expression of RalBP1 selectively impairs NMDAR-dependent LTD.

LTD deficits give rise to corresponding enhancement in potentiation, a metaplastic shift [47]. However, LTP induced by TBS in RalBP1−/− slices (4–7 wk) was not substantially different from that of WT littermates throughout the recording (Figure 10C).

Homosynaptic LTD and depotentiation have many common properties [48]. To induce depotentiation, LFS (1 Hz, 900 stimulations) was delivered to slices (4–7 wk) 5 min after TBS. In contrast to de novo LTD, synaptic depression by LFS after TBS was not different in WT and RalBP1−/− mice (Figure 10D). These results suggest that RalBP1 is involved selectively in NMDAR-dependent de novo LTD, but not in LTP or depotentiation.

### Normal Excitatory Synaptic Transmission at RalBP1−/− CA1 Synapses

**Figure 11:**
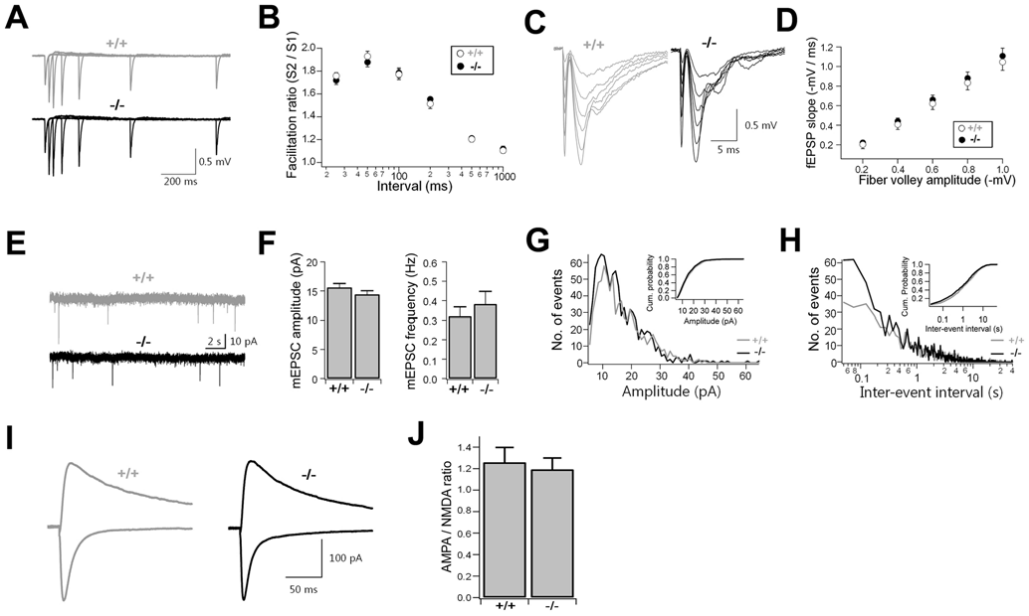
Normal basal synaptic transmission at RalBP1−/− SC-CA1 synapses. (A–B) Normal PPF at RalBP1−/− SC-CA1 synapses. (A) Mean traces of fEPSPs at various stimulus intervals from WT (*n* = 27, 13) and RalBP1−/− slices (*n* = 23, 11). (B) The facilitation ratios plotted as a function of interstimulus intervals. (C–D) Normal synaptic input-output relationship at RalBP1−/− SC-CA1 synapses. Representative traces of fEPSPs at various stimulus intensities (C), and the synaptic input-output relationship for WT (*n* = 11, 10) and RalBP1−/− (*n* = 12, 9) slices (D). (E–H) Normal mEPSCs in CA1 pyramidal neurons. Representative traces (E) and mean amplitude and frequency (F) of mEPSCs. *n* = 23, 5. Amplitude (G) and frequency (H) histogram of mEPSCs. Insets; cumulative probability histograms. (I–J) Normal NMDAR functionality in CA1 pyramidal neurons. (I) Mean traces of AMPAR and NMDAR currents recorded at the holding potential of −70 mV and +40 mV, respectively. *n* = 9, 6 (WT) and 12, 6 (−/−). (J) Mean AMPA/NMDA ratios.

To test whether RalBP1 deficiency affects presynaptic functions at hippocampal SC-CA1 synapses, we examined paired-pulse facilitation (PPF), known to be inversely related to presynaptic release probability. PPF at all interstimulus intervals tested was not changed at RalBP1−/− SC-CA1 synapses (Figure 11A and 11B). In addition, post-tetanic potentiation, another form of short-term plasticity, measured after TBS also appeared normal in RalBP1−/− mice (Figure 10C and 10D).

We next examined the synaptic input-output relationship and spontaneous miniature EPSCs (mEPSCs) to test if RalBP1 deficiency affected excitatory synaptic functions. The relationship between the number of stimulated axons (presynaptic fiber volley) and the slope of postsynaptic fEPSPs at different stimulus intensities was not changed in RalBP1−/− slices (Figure 11C and 11D). Furthermore, we observed no significant difference in the amplitudes or frequencies of mEPSCs between RalBP1−/− and WT mice (Figure 11E–11H). Because mEPSCs and fEPSPs are mainly mediated by AMPARs, we examined NMDAR functionality by measuring the ratio of AMPAR and NMDAR currents (AMPA/NMDA ratio). The stimulation intensity was adjusted to achieve an AMPA-mediated current of ∼150 pA at the holding potential of −70 mV. The AMPA/NMDA ratios measured in RalBP1−/− slices were not different from those of WT littermates (Figure 11I and 11J). Pipette solutions used to measure mEPSCs and AMPA/NMDA ratios contained high concentrations of EGTA (10 mM) to inhibit possible contamination of recordings by currents activated by increases in intracellular Ca^2+^. This would not be expected to affect our interpretations because RalBP1 selectively regulates NMDA-induced AMPAR endocytosis and LTD but not surface AMPAR levels associated with mEPSCs and AMPA/NMDA ratios (Figures 6 and 7). Together, these results suggest that neither AMPA- nor NMDA-mediated excitatory synaptic transmission was affected by the reduced expression of RalBP1 under basal conditions.

## Discussion

### RalBP1 and PSD-95 in NMDAR-Dependent AMPAR Endocytosis and LTD

Our results suggest that RalBP1 is important for NMDAR-dependent AMPAR endocytosis and LTD. In support of this, NMDA treatment rapidly dephosphorylates RalBP1 via PP1 and enhances RalBP1 binding to PSD-95. In addition, RalBP1 knockdown in cultured neurons, or RalBP1 inhibition by overexpression of POB1 CC or phosphomimetic RalBP1 (TE), decreases NMDA-induced GluR2 endocytosis. RalBP1 knockdown in slice culture reduces LTD. Reduced RalBP1 expression in RalBP1−/− mice suppresses LFS-induced LTD. Quantitatively, reduced RalBP1 expression in transfected slices and mice reduced LTD magnitudes by ∼50%–60%, despite that RalBP1 expression was not completely blocked; that is, RalBP1−/− mice have ∼18% of residual RalBP1 expression. RalBP1 knockdown or inhibition in cultured neurons, however, decreased NMDA-induced GluR2 endocytosis by ∼20%. This difference may arise from the use of exogenous GluR2, or chemical (not electrical) LTD induction, in cultured neurons.

How might the RalBP1–PSD-95 interaction promote AMPAR endocytosis during NMDAR-dependent LTD? RalBP1 directly binds AP2 and POB1 [34],[35], which further associate with EH domain-containing endocytic proteins epsin and Eps15 [36]. Therefore, enhanced RalBP1 binding to PSD-95 may bring RalBP1-associated endocytic proteins such as AP2 and POB1 close to PSD-95, which is linked to the complex of TARPs and AMPARs [18]. In support of this possibility, we demonstrated that RalBP1 forms a complex with POB1 and AP2 (α-adaptin) in the brain. The association between RalBP1 and POB1 was particularly strong, to an extent that POB1 is destabilized in RalBP1−/− neurons.

Our data suggest that RalBP1 is rephosphorylated by PKA during the recovery phase (∼1 h) after NMDA treatment. This would dissociate RalBP1 and RalBP1-associated endocytic proteins from PSD-95 and AMPARs, diminishing the drive for AMPAR endocytosis. Consistently, PKA activation markedly reduces NMDA (not AMPA)-induced AMPAR endocytosis, whereas PKA inhibition slightly increases NMDA-induced AMPAR endocytosis [11]. In addition, PKA activation prevents LTD induction and reverses previously established LTD, and PKA inhibition occludes LTD [49].

### RalA in NMDAR-Dependent AMPAR Endocytosis and LTD

In support of the role for RalA in AMPAR endocytosis during NMDAR-dependent LTD, NMDAR activation rapidly induces RalA activation. Activated RalA binds and translocates RalBP1 to dendritic spines. NMDA-induced AMPAR endocytosis is suppressed by knockdown of RalA, and inhibition of RalA by overexpression of RalBD and RalA (S28N). In slice culture, RalBD overexpression suppresses LTD.

How does RalA activation contribute to NMDAR-dependent AMPAR endocytosis? A straightforward possibility is that activated RalA binds and translocates RalBP1 and RalBP1-associated endocytic proteins to synapses, where target AMPARs are located. In addition, RalA-dependent translocation of RalBP1 to synapses might bring RalBP1 close to PSD-95, facilitating their predicted interaction and mediation of NMDAR-dependent AMPAR endocytosis. A previous study has reported that PP1 is recruited to synapses in response to NMDAR activation [50]. Thus, LTD-inducing NMDAR activation would seem to bring both enzyme (PP1) and substrate (RalBP1) together at synapses, enabling their functional interaction. RalA does not seem to affect other RalBP1 functions; in particular, the ability to interact with other proteins such as PSD-95 and POB1 is unchanged by RalA binding to RalBP1 (K.H., M.K., and E.K., unpublished data).

An important question for future study would be to determine how NMDAR activation leads to the activation of RalA. Ras, Rap, and Ca^2+^ are known to act upstream of RalA [29],[30]. Importantly, Rap1 regulates NMDAR-dependent AMPAR endocytosis during LTD via p38 MAPK [42]. In addition, a Drosophila study reported that Rap is more important than Ras for Ral activation [51]. These results suggest that NMDAR might activate RalA via Rap1.

### RalA and RalBP1 Act in Concert to Mediate NMDAR-Dependent AMPAR Endocytosis

Our data indicate that RalA and RalBP1 act together to mediate NMDAR-dependent AMPAR endocytosis. NMDAR activation induces both RalA activation and RalBP1 dephosphorylation. Spine translocation of RalBP1 induced by RalA is further enhanced by NMDA treatment (Figure 4), which results in dephosphorylation of RalBP1. Therefore, synaptic localization of RalBP1 seems to be mediated by a dual mechanism involving the regulated binding of RalBP1 to both RalA and PSD-95; these processes require RalA activation and RalBP1 dephosphorylation, respectively.

Our data also indicate that both RalA and RalBP1 are necessary and sufficient to mediate NMDAR-dependent AMPAR endocytosis. In support of this, RalA activation combined with RalBP1 binding to PSD-95 is sufficient to reduce surface AMPAR levels in the absence of NMDAR activation; it also occludes the NMDA-induced reduction in surface AMPAR levels (Figure 8). However, RalA alone (RalA G23V alone or RalA G23V cotransfected with RalBP1 ΔC) or RalBP1 alone (RalBP1 WT cotransfected with RalA WT) is not sufficient to reduce surface AMPAR levels. In addition, RalA G23V alone does not occlude the NMDA-induced reduction in surface AMPAR levels. The requirement for these two mechanisms—RalA activation and RalBP1 dephosphorylation—in NMDAR-dependent AMPAR endocytosis suggests that these two events may function as a dual-key mechanism that protects against AMPAR endocytosis under conditions in which only a single criterion is fulfilled.

### Hippocalcin, RalBP1, and RalA

Hippocalcin, which binds calcium as well as AP2 (β2 adaptin), is translocated to the synaptic plasma membrane via the calcium-myristoyl switch to mediate NMDAR-dependent AMPAR endocytosis during LTD [52]. RalBP1 is similar to hippocalcin in that it directly interacts with AP2 (μ2 subunit), but it differs from hippocalcin in that it does not have a calcium-sensing activity. Another difference between hippocalcin and RalBP1 is that RalBP1 can be dephosphorylated by NMDAR activation.

An interesting question is whether and how these two pathways (hippocalcin and RalBP1), which are calcium-dependent and phosphatase (PP1)-dependent, respectively, act together to mediate NMDAR-dependent AMPAR endocytosis during LTD. Both hippocalcin and RalBP1 interact with AP2, so it is possible that AP2 may function as a point of crosstalk or convergence between the two pathways. It is conceivable that AP2 and AP2-associated endocytic proteins may be more efficiently translocated to the synaptic plasma membrane by interacting with both hippocalcin and RalBP1.

Lastly, our study has two general implications. Our study provides the first specific mechanism for the general question of how the interaction of activated RalA with RalBP1 is coupled to the endocytosis of target membrane proteins. That is, in our case, the direct interaction of dephosphorylated RalBP1 with a scaffolding protein that is coupled to target membrane proteins. Secondly, our results suggest that scaffolding proteins can switch their functions from the maintenance to regulated endocytosis of interacting membrane proteins. This principle may be applicable to diverse scaffolding proteins that are in association with membrane proteins including receptors, channels, transporters, and adhesion molecules.

In conclusion, our in vitro and in vivo results suggest that the dual and regulated binding of RalBP1 with RalA and PSD-95 mediates AMPAR endocytosis during NMDAR-dependent LTD. Possible directions for future studies include investigation of detailed upstream and downstream mechanisms of this regulated tripartite interaction.

## Materials and Methods

### cDNA Constructs

Full-length RalBP1, human (aa 1–655) and rat (aa 1–647) amplified from brain cDNA libraries (Clontech), and RalBP1 variants (ΔC, aa 1–651 and T653E; human) were subcloned into GW1 (British Biotechnology), pIRES2-EGFP (Clontech), p3XFLAG-CMV-7.1 (Sigma), and YFP-FKBP (from Tobias Meyer). Rat RalBP1 T645A and T645E were subcloned into p3XFLAG-CMV-7.1. Rat RalBP1 R642A was generated using QuickChange kit (Stratagene). A C-terminal region of human RalBP1 (aa 410–655; I655A and T653E) was subcloned into pBHA. POB1 short (aa 1–521; full length; human) was PCR amplified from a cDNA library. POB1 CC (aa 429–521) was subcloned into pEGFP-C1. POB1 long cDNA was kindly gifted from Dr. Blok (Erasmus University, Rotterdam, Netherlands). RalA (full length; mouse) was amplified from a cDNA library and subcloned into pcDNA3.1-HA (Invitrogen). RalA mutants were generated using QuickChange kit. The RalBD (aa 397–518 of human RalBP1) was subcloned into pGEX4T-1 (Amersham Biosciences) and pEGFP-C1. For short hairpin RNA (shRNA) knockdown of RalBP1, pSUPER RalBP1 was generated by annealing oligonucleotides containing nt 666–684 of rat RalBP1 cDNA (U28830; critical 19 nt, GCACGGCATGAAATGTGAA) and subcloning into pSUPER.gfp/neo (OligoEngine). pSUPER RalA was generated using oligonucleotides containing nt 402–420 of rat RalA cDNA (NM_031093; AAGGCAGGTTTCTGTAGAA). pSuper RalA scrambled was generated using oligonucleotides containing the following sequence (GAACGAGTGTCTGTAAGTA). shRNA-resistant RalA rescue construct was generated by introducing point mutations into pcDNA3.1-HA RalA WT using QuickChange kit. The changed nt are indicated in Figure S7. For shRNA-resistant RalBP1 rescue construct, the human version of RalBP1 in GW1 was used. To generate GW1 PSD-95-FRB, full-length rat PSD-95 was first subcloned into YFP-FRB (from Tobias Meyer). From this YFP-PSD-95-FRB plasmid, PSD-95-FRB part was amplified and subcloned into GW1. HA-GluR2 was kindly provided by Dr. Maria Passafaro.

### Antibodies

GST fusion proteins containing human RalBP1 (aa 410–655) and (aa 1–234) were used for immunization (#1403; guinea pig, #1849; guinea pig, respectively). The phospho and non-phospho RalBP1 antibodies (RalBP1-pT645, #1480; RalBP1-T645, #1477; rabbit) were generated using synthetic peptides mimicking the last 10 residues of rat RalBP1 with or without phosphor-threonine at the −2 position (PSKDRKETPI). The POB1 antibody (#1650; guinea pig) was generated using synthetic peptide mimicking the last 15 residues of human POB1 (ALENQLEQLRPVTVL). The following antibodies were purchased: Myc and HA rabbit polyclonal (Santa Cruz), HA mouse monoclonal (Boehringer Mannheim), RalA (BD Biosciences), surface GluR2 mouse monoclonal (Chemicon), α-adaptin, Flag, synaptophysin, and α- and β-tubulin (Sigma).

### Hippocampal Neuron Culture, Transfection, and Immunocytochemistry

Cultured hippocampal neurons were prepared from embryonic day 18 rat brain. Dissociated neurons on poly-L-lysine coated (1 mg/ml) coverslips were placed in Neurobasal medium supplemented with B27 (Invitrogen), 0.5 mM L-glutamine, and penicillin-streptomycin. Cultured neurons were transfected using mammalian transfection kit (Invitrogen) and fixed with 4% paraformaldehyde/sucrose, permeabilized with 0.2% Triton X-100, and incubated with primary and dye-conjugated secondary antibodies.

### RalA-GTP Pulldown Assay

Neurons were lysed in the Ral binding buffer (10% glycerol, 1% Nonidet P-40, 50 mM Tris-HCl, pH 7.4, 200 mM NaCl, 2.5 mM MgCl_2_), followed by pull down by GST-RalBD precoupled to glutathione beads.

### Antibody Feeding Assay

Live neurons expressing HA-GluR2 were incubated with mouse HA antibodies (10 µg/ml) for 10 min at 37°C. After DMEM washing, neurons were returned to conditioned medium containing 20 µM NMDA and incubated at 37°C for 3 min and in the same media without NMDA for 10 min. Neurons were incubated with Cy3 antibodies for surface GluR2, permeabilized, and labeled with Cy5 and FITC antibodies for internalized GluR2 and coexpressed proteins, respectively.

### Surface and Internal GluR2 Labeling

HA-GluR2-expressing neurons were fixed and incubated with rabbit HA antibodies for surface GluR2, followed by permeabilization with 0.2% Triton X-100 and incubation with mouse HA antibodies for internal GluR2. Cy3-, Cy5-, and FITC-conjugated secondary antibodies visualized surface GluR2, internal GluR2, and other coexpressed proteins, respectively.

### Preparation of Subcellular and Postsynaptic Density Fractions

Subcellular rat brain fractions were prepared as described [53]. Briefly, rat brains were homogenized in buffered sucrose (0.32 M sucrose, 4 mM HEPES, 1 mM MgCl_2_, 0.5 mM CaCl_2_, pH 7.3) with freshly added protease inhibitors (this homogenate fraction is H). The homogenate was centrifuged at 900 g for 10 min (the resulting pellet is P1). The resulting supernatant was centrifuged again at 12,000 g for 15 min (the supernatant after this centrifuge is S2). The pellet was resuspended in buffered sucrose and centrifuged at 13,000 g for 15 min (the resulting pellet is P2; crude synaptosome). The S2 fraction was centrifuged at 250,000×g for 2 h (the resulting supernatant is S3, and pellet is P3). The P2 fraction was resuspended in buffered sucrose and added of 9 volume of water]. After homogenization, the homogenate was centrifuged at 33,000 g for 20 min (the resulting pellet is LP1). The resulting supernatant was centrifuged at 250,000×g for 2 h (the resulting supernatant is LS2, and pellet is LP2). PSD fractions were purified as described [54],[55]. To obtain PSD fractions, the synaptosomal fraction was extracted with detergents, once with Triton X-100 (PSD I), twice with Triton X-100 (PSD II), and once with Triton X-100 and once with sarcosyl (PSD III).

### Western Blotting and Coimmunoprecipitation with Stimulated Slices

For Western blot analysis of RalBP1 phosphorylation, homogenates of hippocampal slices were prepared as described previously [12]. Briefly, hippocampal slices were sonicated in resuspension buffer (10 mM sodium phosphate [pH 7.0], 100 mM NaCl, 10 mM sodium pyrophosphate, 50 mM NaF, 1 mM sodium orthovanadate, 5 mM EDTA, 5 mM EGTA, 1 µM okadaic acid, and 10 U/ml aprotinin) and centrifuged at 14,000 g for 10 min at 4°C. The pellets were resuspended in SDS sample loading buffer. For coimmunoprecipitation, hippocampal slices were sonicated in resuspension buffer containing 1% Triton X-100 and 1% saponin, and additionally lyzed for 1 hr at 4°C. After centrifuge at 14,000 g for 10 min at 4°C, supernatants were incubated with antibodies for immunoprecipitation.

### Image Acquisition and Quantification

Z-stack images were acquired using a confocal microscope (LSM510; Zeiss) under the same parameter settings for all scanning. All transfected neurons, with the exception of those with obvious morphological abnormalities, were imaged in an unbiased manner. Image analyses were performed by a researcher blinded to the experimental conditions. Morphometric measurements on randomly selected images were performed using MetaMorph (Universal Imaging). Neuronal areas for surface/internal GluR2 analysis were manually selected.

### Slice Culture, Transfection, and Electrophysiology

Neurons in slice culture were transfected using a gene gun (BioRad) and DNA-coated gold particles. Electrophysiological recordings were performed in solution containing (in mM): NaCl 119, KCl 2.5, CaCl_2_ 4, MgCl_2_ 4, NaHCO_3_ 26, NaH_2_PO_4_ 1, glucose 11, picrotoxin 0.1, and 2-chloradenosine 0.002, at pH 7.4. Patch recording pipettes (3–6 MΩ) contained (in mM): cesium methanesulfonate 115, CsCl 20, HEPES 10, MgCl_2_ 2.5, Na_2_ATP 4, Na_3_GTP 0.4, sodium phosphocreatine 10, and EGTA 0.6, at pH 7.25. Whole-cell recordings were made simultaneously from a pair of CA1 pyramidal neurons (transfected and untransfected) by stimulating presynaptic fibers at 0.2 Hz. Synaptic AMPAR responses were recorded at −70 mV. LTD was induced by pairing 300 pulses at 1 Hz at −45 mV, 15 min after formation of whole-cell configuration. Experiments were blinded in regard to the DNA constructs used.

### Generation of RalBP1 Genetrap Mice

A mouse ES cell line (RRC077, strain 129/Ola) trapped in the RalBP1 gene was provided by Baygenomics. The gene-trap cassette (pGT1lxf) was integrated into a site 15 bp downstream of the 5′ end of the intron 3. The ES cells were injected into blastocysts (C57BL/6J) to generate chimera. Heterozygotes (N1) were backcrossed to C57BL/6J for 4–6 generations. Littermates derived from heterozygous parents were used for all analyses.

### Electrophysiology

Vibratome hippocampal sections (400 µm) were used for voltage-clamp recordings using a MultiClamp 700B amplifier (Axon instruments). Signals were filtered at 2.8 kHz and digitized at 10 kHz. Pipette (2–3 MΩ) contained (in mM): CsMeSO_4_ 100, TEA-Cl 10, NaCl 8, HEPES 10, QX-314-Cl 5, Mg-ATP 2, Na-GTP 0.3, EGTA 10 with pH 7.25, 290 mOsm. Picrotoxin (100 µM) was used to inhibit IPSCs. EPSCs were evoked (0.05 Hz) with ACSF-filled glass pipette (0.3∼0.5 MΩ) placed in stratum radiatum. Mean AMPAR/NMDAR currents were obtained by averaging 30–40 traces recorded at the holding potential of −70 mV or +40 mV. NMDAR currents were isolated by NBQX (20 µM). mEPSCs were recorded at a holding potential of −60 mV with TTX (1 µM) in ACSF. For extracellular recordings, submerged slices were evoked at 0.05 Hz with a stimulation intensity that yields a half-maximal response. Homosynaptic LTD was induced by delivering 900 stimulations (1 Hz), and LTP was induced by four episodes (0.1 Hz) of TBS. mGluR-dependent LTD was induced by paired-pulse stimulation (50 ms interstimulus interval) repeated at 1 Hz for 15 min (for Figure 5) [56], or by DHPG application (for Figure 10). Data were analyzed by using custom macros written in Igor (WaveMetrics).

## Supporting Information

Figure S1 **Expression patterns of RalBP1, RalA, and POB1 mRNAs.** Distribution patterns of mRNAs for RalBP1 (A), RalA (B), and POB1 (C) in adult (6 wk) rat brain revealed by in situ hybridization analysis. OB, olfactory bulb; Ctx, cortex; Hc, hippocampus; Cb, cerebellum.
(2.08 MB TIF)

Figure S2 **Expression patterns of RalBP1, RalA, and POB1 proteins.** (A, B) RalBP1 and POB1 proteins expressed in rat brain have molecular masses similar to those expressed in heterologous cells. Note that the molecular weights of RalBP1 and POB1 expressed in the brain are identical and similar, respectively, to those expressed in HEK293T cells. POB1 short and long, short and long variants of POB1; Untrans, untransfected; P2, crude synaptosomal fraction; S2, supernatant after P2 precipitation. A nonspecific POB1 band, which is not detected by independent POB1 antibodies, is indicated by an asterisk. (C) Tissue distribution patterns of RalBP1, RalA, and POB1 proteins in adult rat. Note that RalBP1, RalA, and POB1 are most abundantly expressed in the brain. PSD-95 was blotted for comparison. Sk., skeletal. (D) Expression of RalBP1, RalA, and POB1 proteins in different brain regions. Homogenates of adult (6 wk) rat brain regions were immunoblotted for RalBP1, RalA, POB1, PSD-95, and β-tubulin (control). St, striatum; R, the rest of the brain. (E) Expression patterns of RalBP1, RalA, and POB1 proteins during rat brain development. Whole homogenates of rat brains at the indicated developmental stages were immunoblotted for RalBP1, RalA, POB1, α-adaptin, PSD-95, and β-tubulin (control). E, embryonic day; P, postnatal day. (F) Distribution of RalBP1 and RalA in subcellular fractions of rat brains at P21 and 6 wk. SynPhy, synaptophysin (control); H, homogenates; S3, cytosol; P3, light membranes; LP1, synaptosomal membranes; LS2, synaptosomal cytosol; LP2, synaptic vesicle-enriched fraction. (G) RalBP1 and RalA are not tightly associated with the PSD. PSD fractions of adult (6 wk) rat brain extracted with Triton X-100 once (PSD I), Triton X-100 twice (PSD II), or with Triton X-100 and the strong detergent sarcosyl (PSD III), were immunoblotted with the indicated antibodies.
(1.08 MB TIF)

Figure S3 **RalBP1 translocated to spines by RalAG23V significantly, but not Completely, colocalizes with PSD-95.** Neurons transfected with RalA G23V+RalBP1 (untagged), or RalA G23V+RalBP1 (untagged)+PSD-95 (untagged) (DIV 18–19), were stained for RalBP1 and PSD-95 (endogenous and exogenous).
(0.50 MB TIF)

Figure S4 **Expression of RalA and RalBP1 in cultured neurons does not affect the head area of dendritic spines.** Cultured neurons transfected with RalA (WT or mutants)+RalBP1 (WT or mutants)+EGFP (DIV 17–18) were immunostained for EGFP and RalBP1. The head area of dendritic spines was measured from EGFP images.
(0.90 MB TIF)

Figure S5 **RalA forms a ternary complex with RalBP1 and PSD-95 and recruits PSD-95 to the plasma membrane via RalBP1.** (A) RalA WT and RalA (S28N; dominant negative) do not form a complex with RalBP1 (the RalBD domain of RalBP1) in heterologous cells, whereas constitutively active RalA (G23V) does. HEK293T cell lysates doubly transfected with HA-RalA (WT, G23V, or S28N) and EGFP-RalBD were immunoprecipitated with EGFP antibodies and immunoblotted with HA and EGFP antibodies. (B) RalA G23V, but not RalA S28N, forms a ternary complex with RalBP1 and PSD-95 in heterologous cells. Lysates of HEK293T cells triply transfected with HA-RalA (G23V or S28N), RalBP1, and PSD-95 were immunoprecipitated with HA antibodies and immunoblotted with the indicated antibodies. (C) RalA G23V translocates PSD-95 to the plasma membrane via RalBP1. HEK293T cells were transfected with the indicated combinations HA-RalA G23V, RalBP1 (WT or ΔC), and PSD-95, followed by immunofluorescence staining. Note that RalG23V fails to translocate PSD-95 to the plasma membrane when RalBP1 ΔC that lacks PSD-95 binding is used, instead of WT RalBP1.
(1.16 MB TIF)

Figure S6 **Characterization of RalA and RalBP1 shRNA constructs in heterologous cells and cultured neurons.** (A, B) shRNA-mediated knockdown of RalA and RalBP1 in heterologous cells. HEK293T cells were doubly transfected with HA-RalA+pSUPER RalA (sh-RalA), HA-RalA+pSUPER alone (sh-vec; control), or HA-RalA+pSUPER RalA scrambled (sh-RalA SC; control). For RalBP1, cells were doubly transfected with Flag-RalBP1+pSUPER RalBP1 (sh-RalBP1), or Flag-RalBP1+sh-vec. Expression levels of proteins were measured by immunoblotting of the HEK293T cell lysates with HA (for RalA), Flag (for RalBP1), EGFP (for shRNAs), and α-tubulin (loading control) antibodies. The band intensity in the knockdown lanes was normalized to that of sh-vec controls. Mean±SEM (sh-RalA, 0.14±0.11, *n* = 3, * *p*<0.05; sh-RalA SC, 1.01±0.28, *n* = 3, *p* = 0.82; sh-RalBP1, 0.22±0.02, *n* = 3, *** *p*<0.001, Student's *t*-test). (C, D) shRNA-mediated knockdown of RalA and RalBP1 in cultured neurons. Cultured hippocampal neurons were transfected with HA-RalA+sh-RalA/sh-vec, or Flag-RalBP1+sh-RalBP1/sh-vec (DIV 12–15). Expression levels of the target proteins were measured by visualizing the transfected neurons with HA (for RalA), RalBP1, and EGFP (for shRNA) antibodies. Average fluorescence intensities of RalA and RalBP1 in the cell body area were quantified and normalized to sh-vec controls. Mean±SEM (sh-RalA, 0.23±0.01, *n* = 6, ** *p*<0.01; sh-RalBP1, 0.10±0.01, *n* = 7, * *p*<0.05, Student's *t*-test).
(0.97 MB TIF)

Figure S7 **Coexpression of shRNA-resistant RalA and RalBP1 rescues reduced GluR2 endocytosis by RalA and RalBP1 shRNAs.** (A, B) Characterization of shRNA-resistant RalA (RalA res.) and RalBP1 (RalBP1 res.) rescue constructs in heterologous cells. Nucleotide changes in the target 19-bp region of shRNAs in RalA res. and RalBP1 res. are underlined. HEK293T cells were doubly transfected with HA-RalA res.+sh-RalA or sh-vec (A), or RalBP1 res.+sh-RalBP1 or sh-vec (B). Protein expression levels were measured by immunoblotting of the HEK293T cell lysates with HA (for RalA), RalBP1, and EGFP (for shRNAs) antibodies. The band intensities in the knockdown lanes were normalized to those in the control lanes (sh-vec). (C, D) Characterization of shRNA-resistant RalA and RalBP1 rescue constructs in cultured neurons. Cultured hippocampal neurons (DIV 12–15) were transfected with HA-RalA res.+sh-RalA/sh-vec, or RalBP1 res.+sh-RalBP1/sh-vec. Protein expression levels were measured by visualizing the transfected neurons with HA (for RalA), RalBP1, and EGFP (for shRNA) antibodies. Average fluorescence intensities of RalA and RalBP1 in the cell body area were quantified and normalized to sh-vec controls. (E, F) Coexpression of shRNA-resistant RalA or RalBP1 rescues the effects of RalA and RalBP1 shRNAs (reduction in NMDA-induced GluR2 endocytosis). Cultured neurons doubly transfected with HA-GluR2+sh-RalA/sh-RalBP1/sh-vec, or triply with HA-GluR2+sh-RalA/sh-RalBP1+RalA res./RalBP1 res. (DIV 16–20), were subjected to the antibody feeding assay under NMDA treatment condition (20 µM, 3 min). The internalization index was normalized to sh-vec control. *n* = 15–30, * *p*<0.05, ANOVA.
(1.64 MB TIF)

Figure S8 **Effects of RalA (WT and mutants) on NMDA-induced and basal GluR2 Endocytosis, and surface GluR2 levels.** (A) RalA S28N (dominant negative; constitutively in the GDP-bound state) and RalA GVDN (a RalA G23V mutant with weakened RalBP1 binding), but not RalA WT and RalA G23V (constitutively active), reduce NMDA-induced GluR2 endocytosis. Cultured neurons transfected with HA-GluR2 alone, or HA-GluR2+RalA WT or mutants (DIV 16–18), were subject to the antibody feeding assay under NMDA (20 µM, 3 min) treatment condition. The internalization index was normalized to the GluR2 alone control. *n* = 19–27, * *p*<0.05, Student's *t*-test. (B) RalA S28N and RalA GVDN do not affect basal GluR2 endocytosis. Transfected neurons were subject to the antibody feeding assay in the absence of NMDA treatment. The internalization index was normalized to GluR2 alone. *n* = 10–17. (C) RalA S28N, but not other RalA types (WT and mutants), reduces surface GluR2 levels. Transfected neurons were measured for the steady-state surface-to-internal ratio of GluR2. *n* = 19–29, * *p*<0.05, Student's *t*-test.
(2.41 MB TIF)

Figure S9 **Inhibition of active RalA by RalA S28N or knockdown of RalA does not affect GluR2 recycling.** (A) Inhibition of active RalA by overexpression of RalA S28N does not affect GluR2 recycling. Cultured neurons transfected with HA-GluR2+RalA (WT or S28N)+EGFP (DIV 16–20) were subjected to the receptor recycling assay (see Text S1 for details). The surface-to-internal ratios of GluR2 were measured at the indicated time points after acid strip, and normalized to those of 0 min controls. *n* = 15–20, * *p*<0.05, Student's *t*-test. The normalized ratios of RalA WT and RalA S28N at 20 min were not significantly different (*p* = 0.35, Student's *t*-test). (B) RalA knockdown does not affect GluR2 recycling. Cultured neurons doubly transfected with HA-GluR2+sh-RalA or sh-vec (DIV 16–20) were subjected to the receptor recycling assay. *n* = 25–27, *** *p*<0.001, Student's *t*-test. The normalized ratios of sh-RalA and sh-vec at 20 min were not significantly different (*p* = 0.08, Student's *t*-test).
(1.25 MB TIF)

Figure S10 **Distribution patterns of RalBP1-β-geo fusion proteins in mouse brain regions.** (A–D) Widespread distribution patterns of RalBP1-β-geo fusion proteins in mouse brain regions. Forebrain (A, B) and cerebellum (C, D) sections prepared from 4-wk-old RalBP1+/+ and RalBP1+/− littermates were stained by X-gal. Note that the lack of X-gal staining in WT brain indicates the specific expression of RalBP1-β-geo fusion proteins in RalBP1+/− slices. (E–L) High-magnification images of RalBP1-β-geo fusion protein expression in RalBP1+/− brain regions including hippocampus (E), cerebral cortex (F), retrosplenial area (G), amygdala (H), thalamus (I), cerebellum (J), and brainstem (K and L). RSP, retrosplenial area; BLA, basolateral amygdalar nucleus; PIR, piriform area; LD, lateral dorsal nucleus of thalamus; TH, thalamus; PGRN, paragigantocellular reticular nucleus in the medulla; SPVI, spinal nucleus of the trigeminal in the medulla; IP, interposed nucleus in the deep cerebellar nuclei; SPIV, spinal vestibular nucleus in the junctional area between medulla and pons.
(3.96 MB TIF)

Text S1 **Supplementary** **materials and methods** **.**
(0.03 MB DOC)

## Abbreviations

AMPAR: AMPA receptor
CA1: CA1 pyramidal cell
EPSC: excitatory postsynaptic current
GVDN: G23VD49N
LFS: low-frequency electrical stimulation
LTD: long-term depression
mEPSCs: miniature EPSCs
mGluR: metabotropic glutamate receptor
NMDAR: NMDA receptor
PKA: protein kinase A
POB1 CC: CC domain of POB1
PP1: protein phosphatase 1
PP2A: protein phosphatase 2A
PPF: paired-pulse facilitation
PP-LFS: paired-pulse LFS
RalBD: Ral binding domain of RalBP1
SC: Schaffer collateral
shRNA: short hairpin RNA
TBS: theta-burst stimulation
WT: wild-type
