# Supplementary figures and images for: Regulated RalBP1 Binding to RalA and PSD-95 Controls AMPA Receptor Endocytosis and LTD
Source: PLoS Biol. 2009 Sep 8;7(9):e1000187. doi: 10.1371/journal.pbio.1000187 (PMC2730530; doi:10.1371/journal.pbio.1000187)

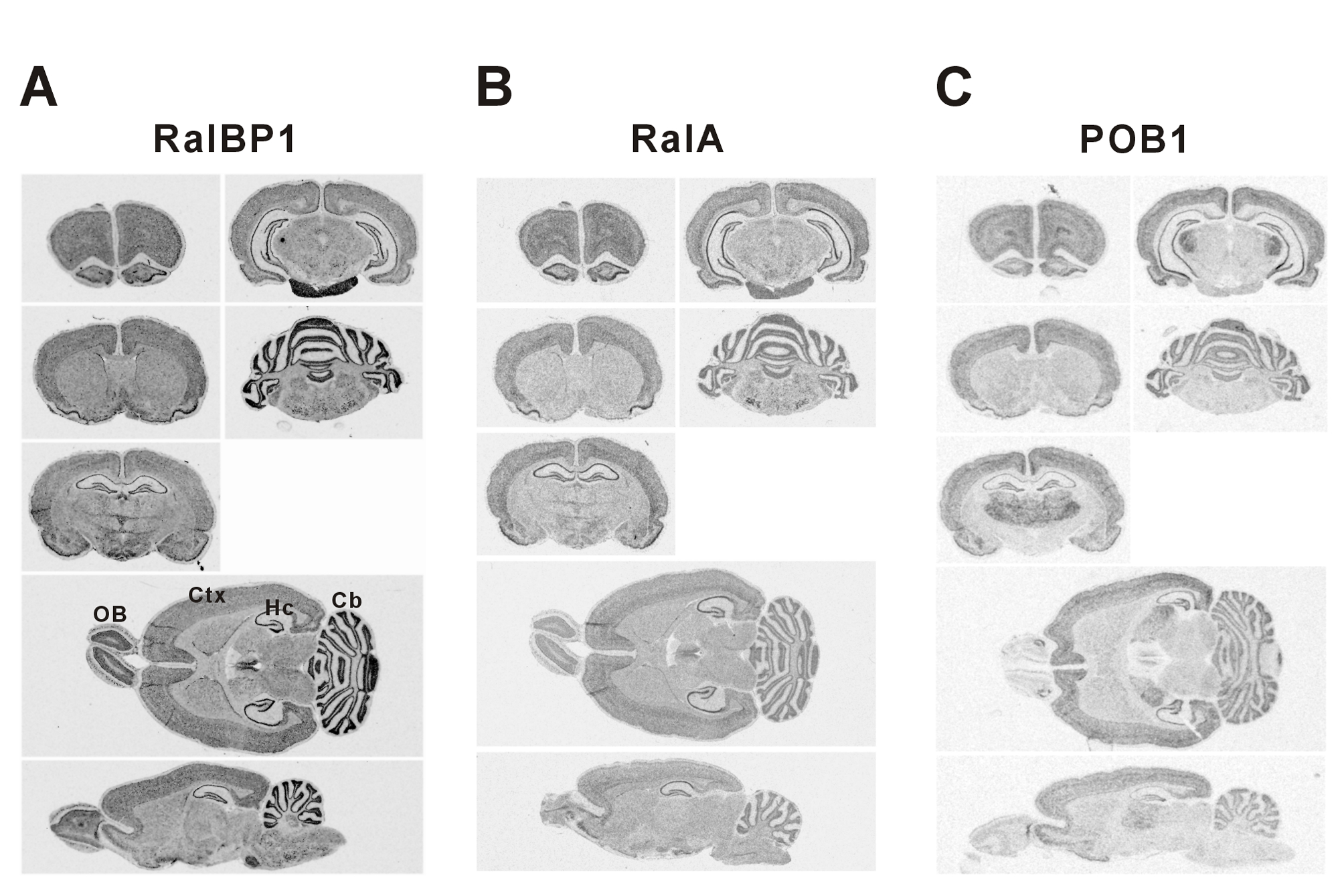

Supplement: Figure S1 — Expression patterns of RalBP1, RalA, and POB1 mRNAs. Distribution patterns of mRNAs for RalBP1 (A), RalA (B), and POB1 (C) in adult (6 wk) rat brain revealed by in situ hybridization analysis. OB, olfactory bulb; Ctx, cortex; Hc, hippocampus; Cb, cerebellum. (2.08 MB TIF) [file pbio.1000187.s001.tif]

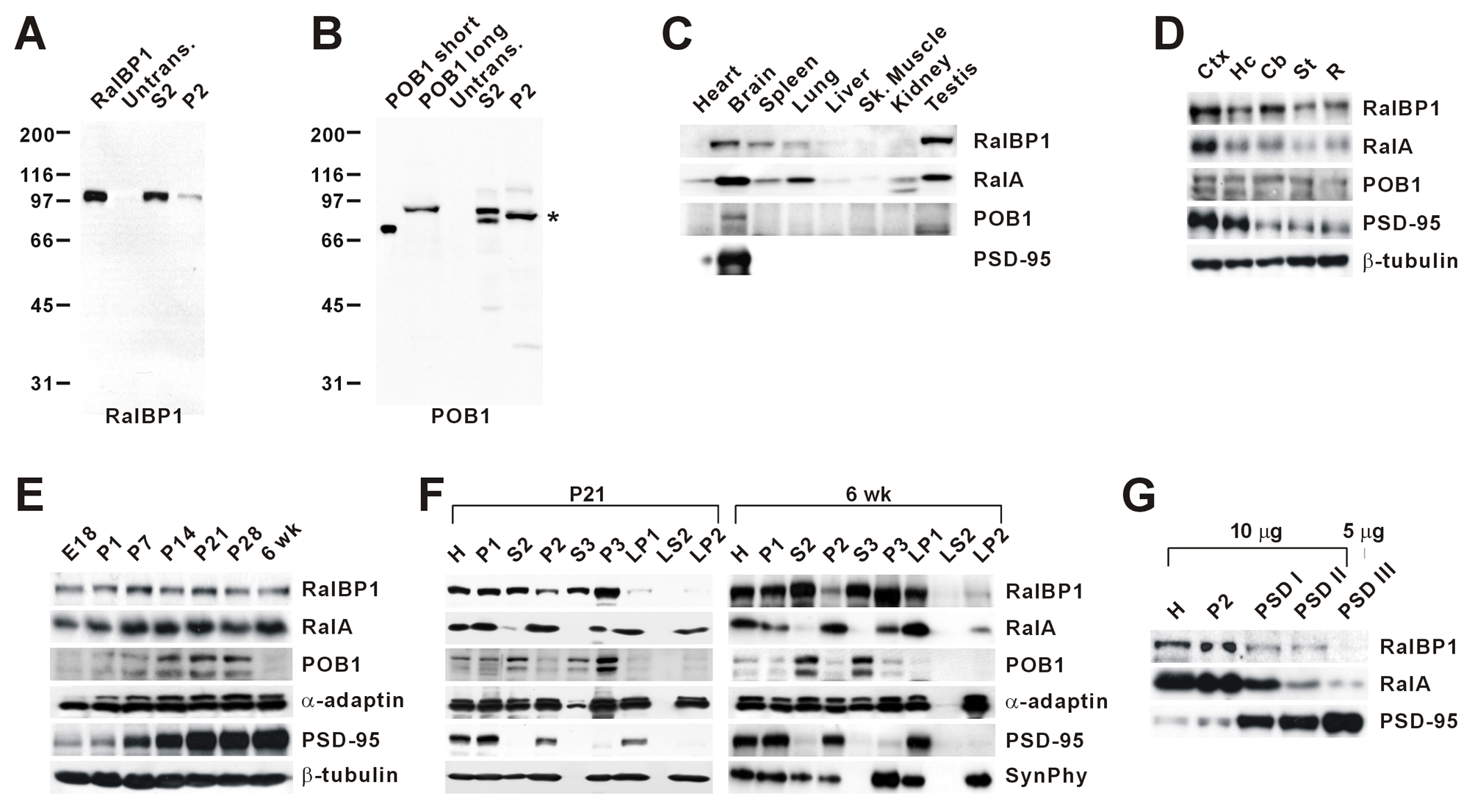

Supplement: Figure S2 — Expression patterns of RalBP1, RalA, and POB1 proteins. (A, B) RalBP1 and POB1 proteins expressed in rat brain have molecular masses similar to those expressed in heterologous cells. Note that the molecular weights of RalBP1 and POB1 expressed in the brain are identical and similar, respectively, to those expressed in HEK293T cells. POB1 short and long, short and long variants of POB1; Untrans, untransfected; P2, crude synaptosomal fraction; S2, supernatant after P2 precipitation. A nonspecific POB1 band, which is not detected by independent POB1 antibodies, is indicated by an asterisk. (C) Tissue distribution patterns of RalBP1, RalA, and POB1 proteins in adult rat. Note that RalBP1, RalA, and POB1 are most abundantly expressed in the brain. PSD-95 was blotted for comparison. Sk., skeletal. (D) Expression of RalBP1, RalA, and POB1 proteins in different brain regions. Homogenates of adult (6 wk) rat brain regions were immunoblotted for RalBP1, RalA, POB1, PSD-95, and β-tubulin (control). St, striatum; R, the rest of the brain. (E) Expression patterns of RalBP1, RalA, and POB1 proteins during rat brain development. Whole homogenates of rat brains at the indicated developmental stages were immunoblotted for RalBP1, RalA, POB1, α-adaptin, PSD-95, and β-tubulin (control). E, embryonic day; P, postnatal day. (F) Distribution of RalBP1 and RalA in subcellular fractions of rat brains at P21 and 6 wk. SynPhy, synaptophysin (control); H, homogenates; S3, cytosol; P3, light membranes; LP1, synaptosomal membranes; LS2, synaptosomal cytosol; LP2, synaptic vesicle-enriched fraction. (G) RalBP1 and RalA are not tightly associated with the PSD. PSD fractions of adult (6 wk) rat brain extracted with Triton X-100 once (PSD I), Triton X-100 twice (PSD II), or with Triton X-100 and the strong detergent sarcosyl (PSD III), were immunoblotted with the indicated antibodies. (1.08 MB TIF) [file pbio.1000187.s002.tif]

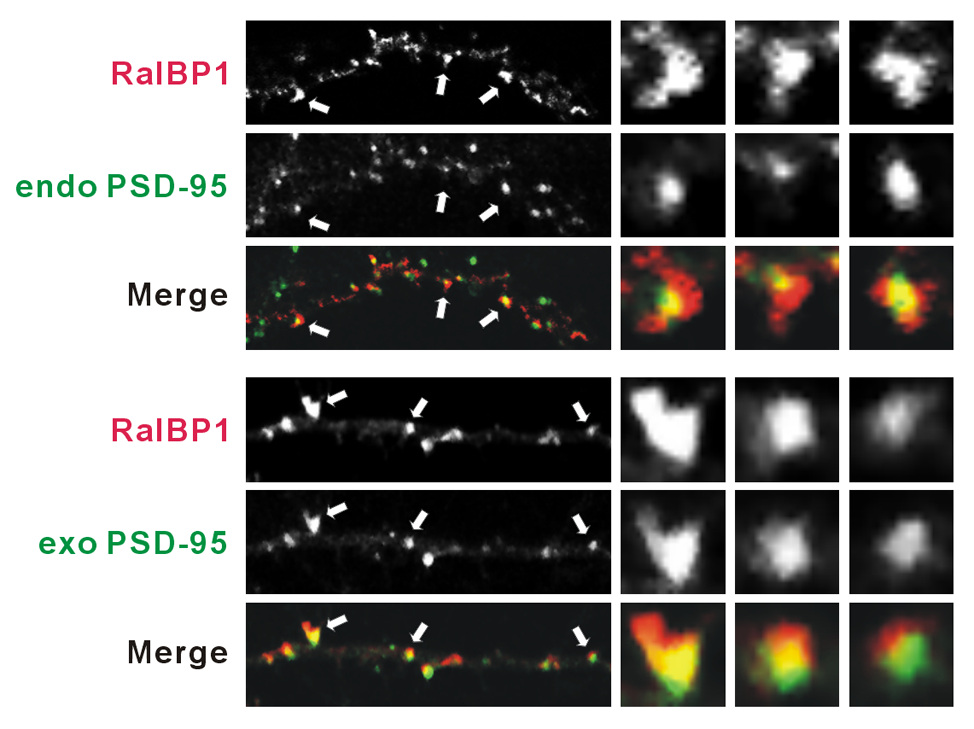

Supplement: Figure S3 — RalBP1 translocated to spines by RalAG23V significantly, but not Completely, colocalizes with PSD-95. Neurons transfected with RalA G23V+RalBP1 (untagged), or RalA G23V+RalBP1 (untagged)+PSD-95 (untagged) (DIV 18–19), were stained for RalBP1 and PSD-95 (endogenous and exogenous). (0.50 MB TIF) [file pbio.1000187.s003.tif]

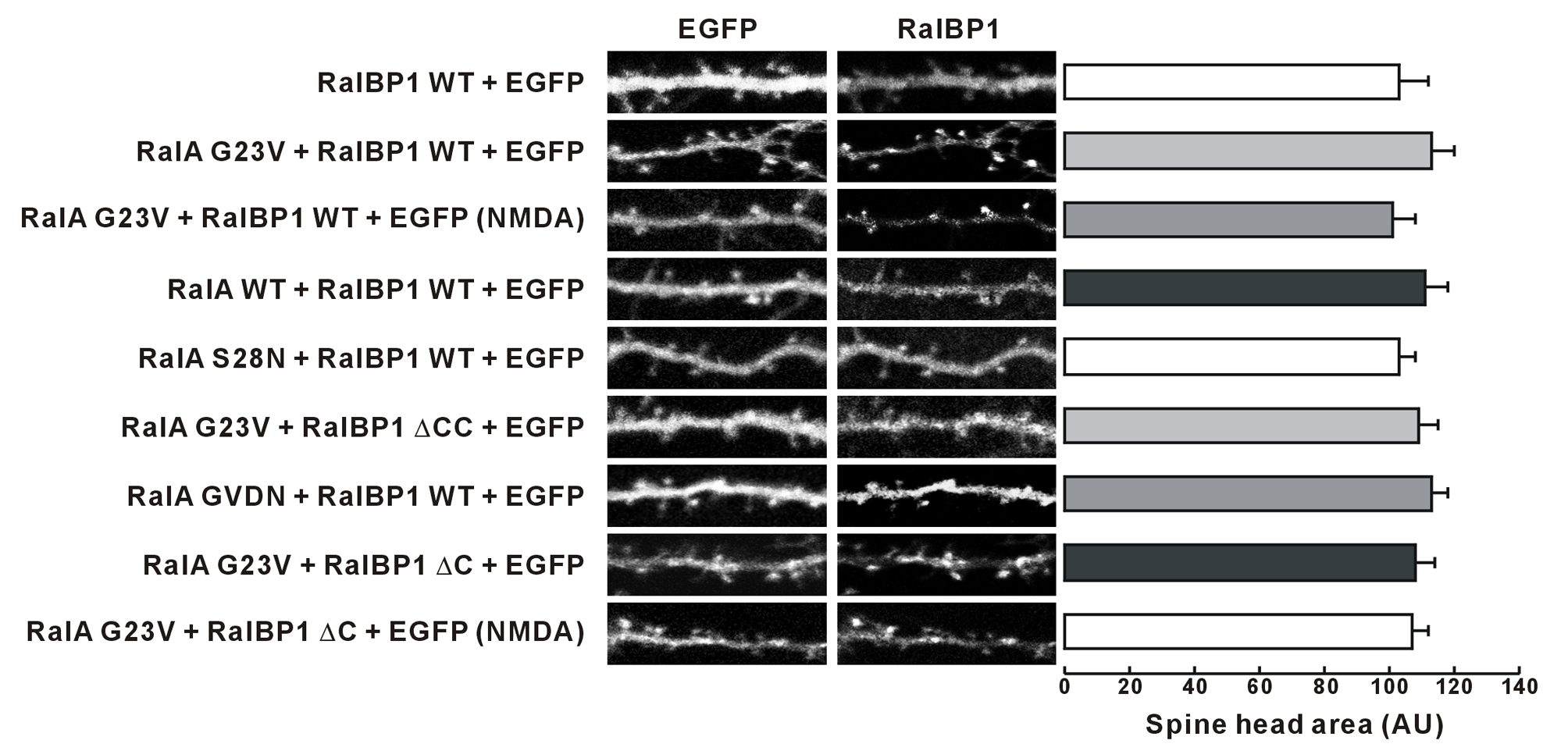

Supplement: Figure S4 — Expression of RalA and RalBP1 in cultured neurons does not affect the head area of dendritic spines. Cultured neurons transfected with RalA (WT or mutants)+RalBP1 (WT or mutants)+EGFP (DIV 17–18) were immunostained for EGFP and RalBP1. The head area of dendritic spines was measured from EGFP images. (0.90 MB TIF) [file pbio.1000187.s004.tif]

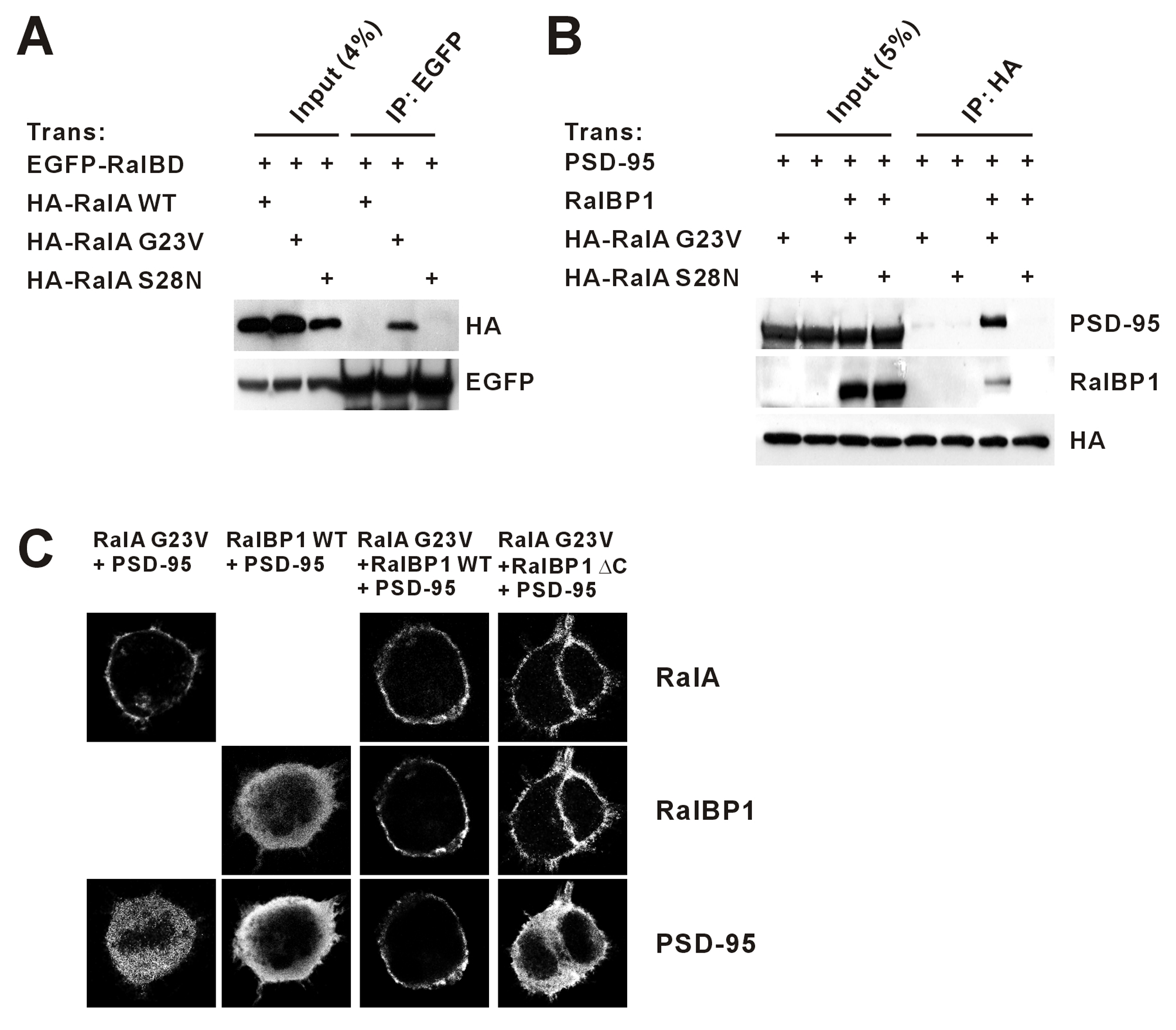

Supplement: Figure S5 — RalA forms a ternary complex with RalBP1 and PSD-95 and recruits PSD-95 to the plasma membrane via RalBP1. (A) RalA WT and RalA (S28N; dominant negative) do not form a complex with RalBP1 (the RalBD domain of RalBP1) in heterologous cells, whereas constitutively active RalA (G23V) does. HEK293T cell lysates doubly transfected with HA-RalA (WT, G23V, or S28N) and EGFP-RalBD were immunoprecipitated with EGFP antibodies and immunoblotted with HA and EGFP antibodies. (B) RalA G23V, but not RalA S28N, forms a ternary complex with RalBP1 and PSD-95 in heterologous cells. Lysates of HEK293T cells triply transfected with HA-RalA (G23V or S28N), RalBP1, and PSD-95 were immunoprecipitated with HA antibodies and immunoblotted with the indicated antibodies. (C) RalA G23V translocates PSD-95 to the plasma membrane via RalBP1. HEK293T cells were transfected with the indicated combinations HA-RalA G23V, RalBP1 (WT or ΔC), and PSD-95, followed by immunofluorescence staining. Note that RalG23V fails to translocate PSD-95 to the plasma membrane when RalBP1 ΔC that lacks PSD-95 binding is used, instead of WT RalBP1. (1.16 MB TIF) [file pbio.1000187.s005.tif]

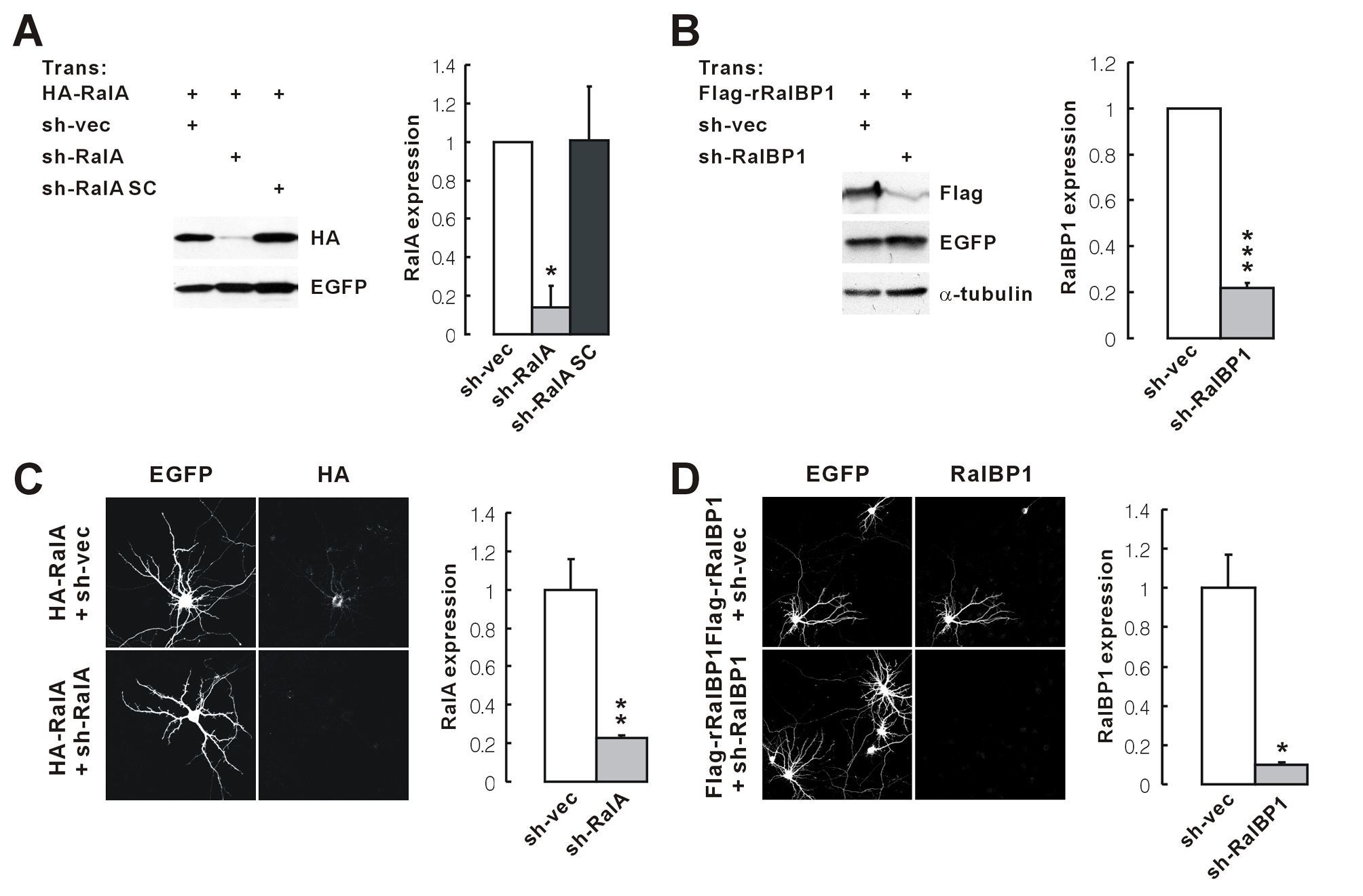

Supplement: Figure S6 — Characterization of RalA and RalBP1 shRNA constructs in heterologous cells and cultured neurons. (A, B) shRNA-mediated knockdown of RalA and RalBP1 in heterologous cells. HEK293T cells were doubly transfected with HA-RalA+pSUPER RalA (sh-RalA), HA-RalA+pSUPER alone (sh-vec; control), or HA-RalA+pSUPER RalA scrambled (sh-RalA SC; control). For RalBP1, cells were doubly transfected with Flag-RalBP1+pSUPER RalBP1 (sh-RalBP1), or Flag-RalBP1+sh-vec. Expression levels of proteins were measured by immunoblotting of the HEK293T cell lysates with HA (for RalA), Flag (for RalBP1), EGFP (for shRNAs), and α-tubulin (loading control) antibodies. The band intensity in the knockdown lanes was normalized to that of sh-vec controls. Mean±SEM (sh-RalA, 0.14±0.11, n = 3, * p<0.05; sh-RalA SC, 1.01±0.28, n = 3, p = 0.82; sh-RalBP1, 0.22±0.02, n = 3, *** p<0.001, Student's t-test). (C, D) shRNA-mediated knockdown of RalA and RalBP1 in cultured neurons. Cultured hippocampal neurons were transfected with HA-RalA+sh-RalA/sh-vec, or Flag-RalBP1+sh-RalBP1/sh-vec (DIV 12–15). Expression levels of the target proteins were measured by visualizing the transfected neurons with HA (for RalA), RalBP1, and EGFP (for shRNA) antibodies. Average fluorescence intensities of RalA and RalBP1 in the cell body area were quantified and normalized to sh-vec controls. Mean±SEM (sh-RalA, 0.23±0.01, n = 6, ** p<0.01; sh-RalBP1, 0.10±0.01, n = 7, * p<0.05, Student's t-test). (0.97 MB TIF) [file pbio.1000187.s006.tif]

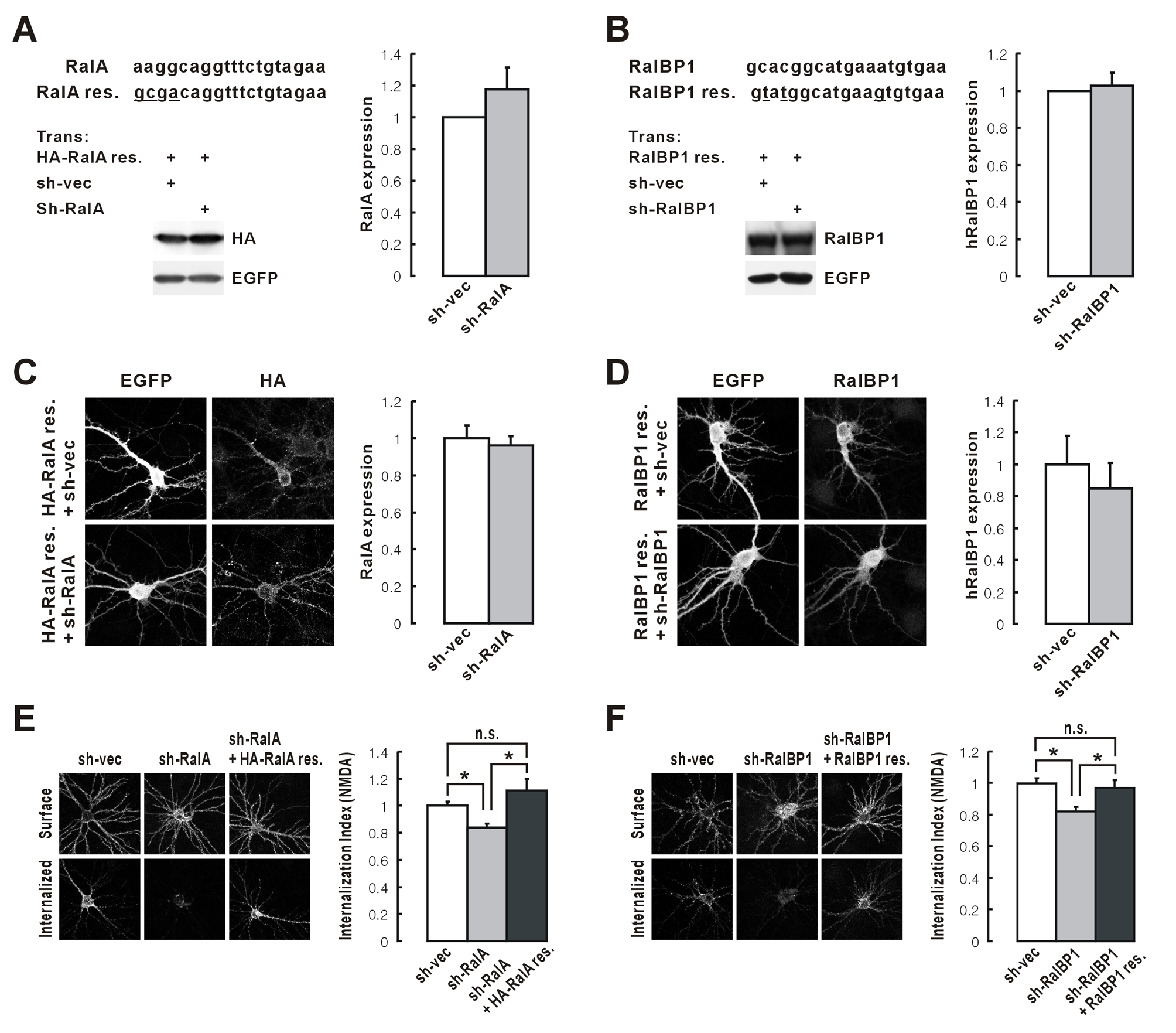

Supplement: Figure S7 — Coexpression of shRNA-resistant RalA and RalBP1 rescues reduced GluR2 endocytosis by RalA and RalBP1 shRNAs. (A, B) Characterization of shRNA-resistant RalA (RalA res.) and RalBP1 (RalBP1 res.) rescue constructs in heterologous cells. Nucleotide changes in the target 19-bp region of shRNAs in RalA res. and RalBP1 res. are underlined. HEK293T cells were doubly transfected with HA-RalA res.+sh-RalA or sh-vec (A), or RalBP1 res.+sh-RalBP1 or sh-vec (B). Protein expression levels were measured by immunoblotting of the HEK293T cell lysates with HA (for RalA), RalBP1, and EGFP (for shRNAs) antibodies. The band intensities in the knockdown lanes were normalized to those in the control lanes (sh-vec). (C, D) Characterization of shRNA-resistant RalA and RalBP1 rescue constructs in cultured neurons. Cultured hippocampal neurons (DIV 12–15) were transfected with HA-RalA res.+sh-RalA/sh-vec, or RalBP1 res.+sh-RalBP1/sh-vec. Protein expression levels were measured by visualizing the transfected neurons with HA (for RalA), RalBP1, and EGFP (for shRNA) antibodies. Average fluorescence intensities of RalA and RalBP1 in the cell body area were quantified and normalized to sh-vec controls. (E, F) Coexpression of shRNA-resistant RalA or RalBP1 rescues the effects of RalA and RalBP1 shRNAs (reduction in NMDA-induced GluR2 endocytosis). Cultured neurons doubly transfected with HA-GluR2+sh-RalA/sh-RalBP1/sh-vec, or triply with HA-GluR2+sh-RalA/sh-RalBP1+RalA res./RalBP1 res. (DIV 16–20), were subjected to the antibody feeding assay under NMDA treatment condition (20 µM, 3 min). The internalization index was normalized to sh-vec control. n = 15–30, * p<0.05, ANOVA. (1.64 MB TIF) [file pbio.1000187.s007.tif]

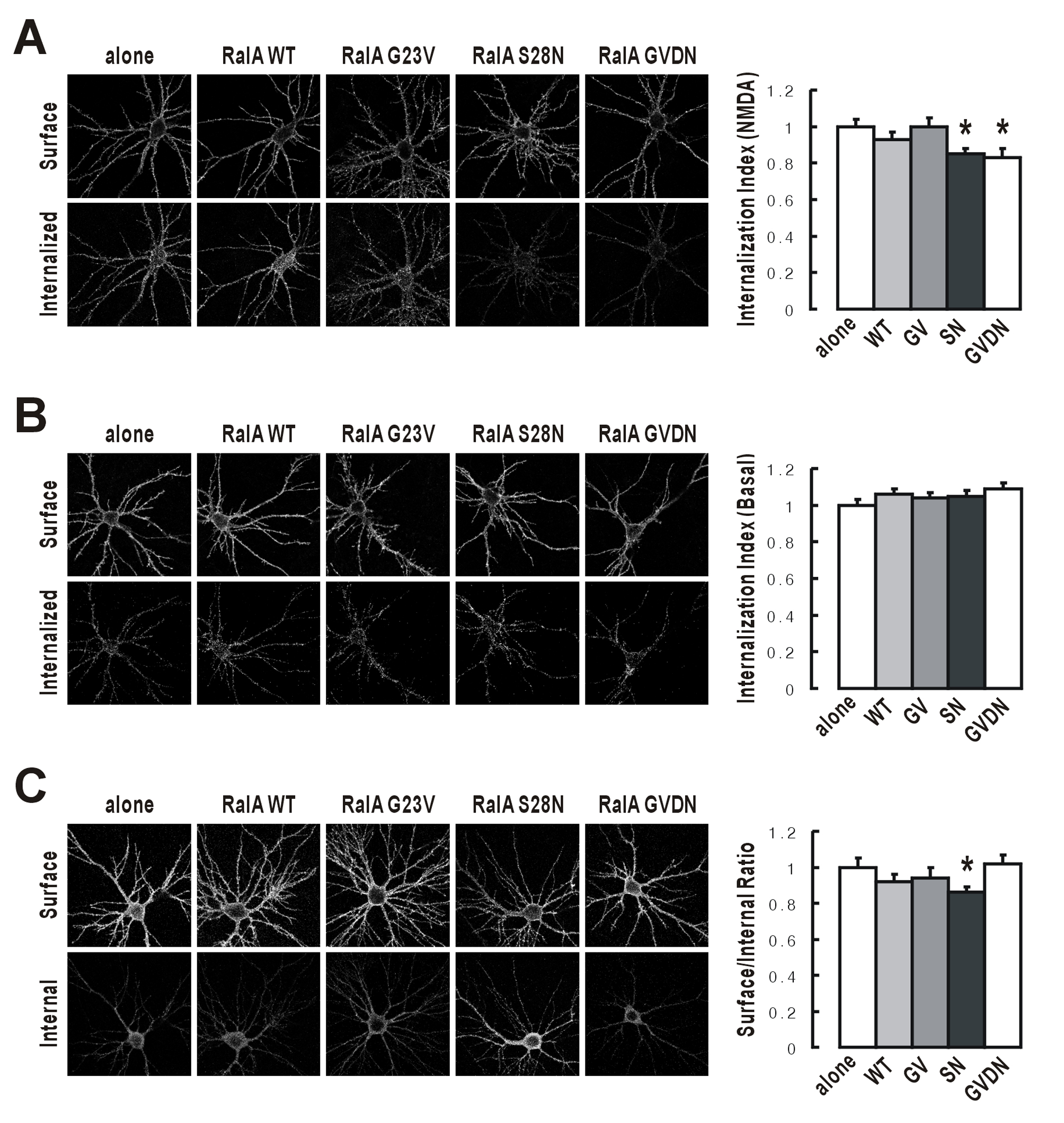

Supplement: Figure S8 — Effects of RalA (WT and mutants) on NMDA-induced and basal GluR2 Endocytosis, and surface GluR2 levels. (A) RalA S28N (dominant negative; constitutively in the GDP-bound state) and RalA GVDN (a RalA G23V mutant with weakened RalBP1 binding), but not RalA WT and RalA G23V (constitutively active), reduce NMDA-induced GluR2 endocytosis. Cultured neurons transfected with HA-GluR2 alone, or HA-GluR2+RalA WT or mutants (DIV 16–18), were subject to the antibody feeding assay under NMDA (20 µM, 3 min) treatment condition. The internalization index was normalized to the GluR2 alone control. n = 19–27, * p<0.05, Student's t-test. (B) RalA S28N and RalA GVDN do not affect basal GluR2 endocytosis. Transfected neurons were subject to the antibody feeding assay in the absence of NMDA treatment. The internalization index was normalized to GluR2 alone. n = 10–17. (C) RalA S28N, but not other RalA types (WT and mutants), reduces surface GluR2 levels. Transfected neurons were measured for the steady-state surface-to-internal ratio of GluR2. n = 19–29, * p<0.05, Student's t-test. (2.41 MB TIF) [file pbio.1000187.s008.tif]

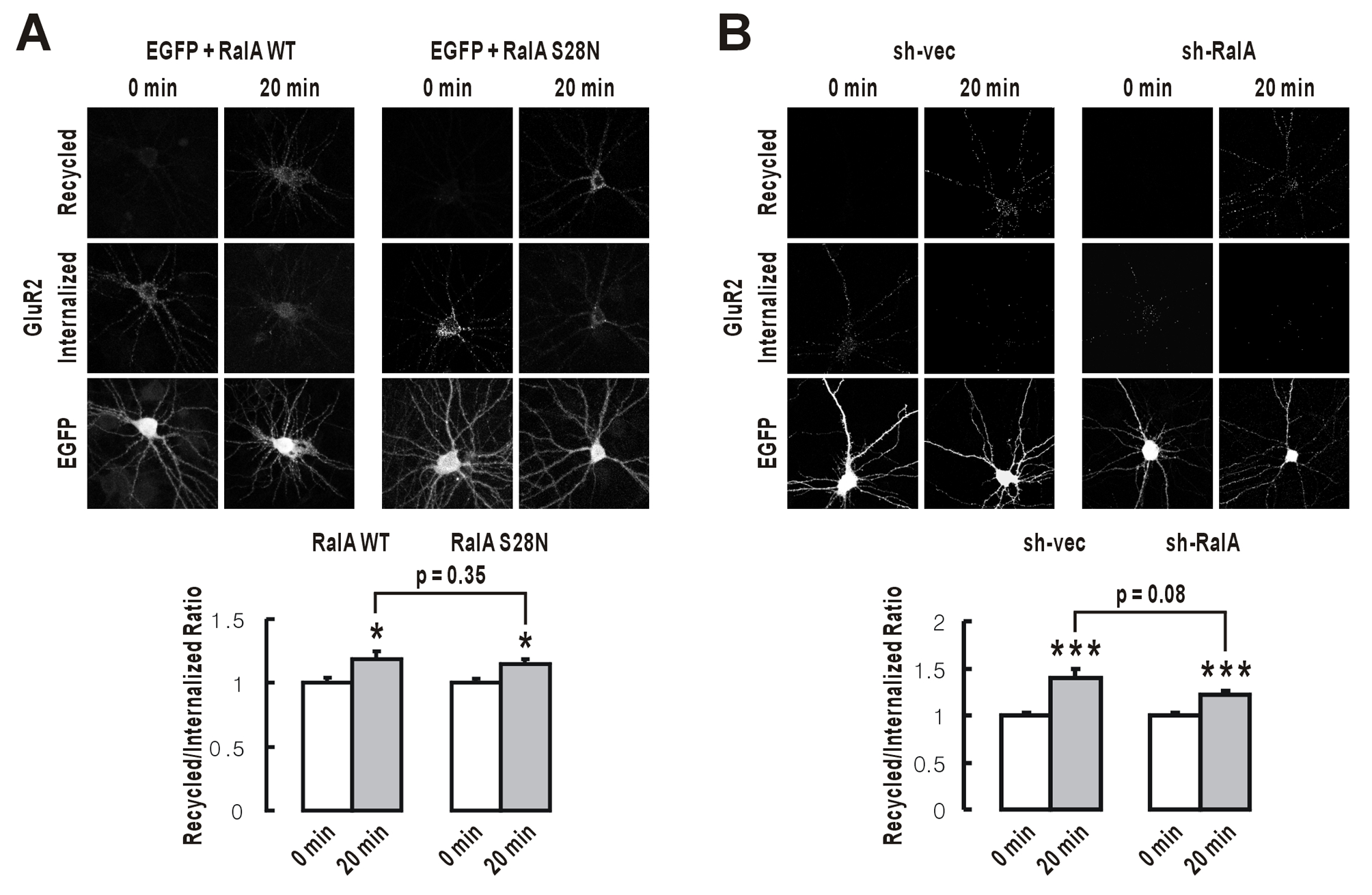

Supplement: Figure S9 — Inhibition of active RalA by RalA S28N or knockdown of RalA does not affect GluR2 recycling. (A) Inhibition of active RalA by overexpression of RalA S28N does not affect GluR2 recycling. Cultured neurons transfected with HA-GluR2+RalA (WT or S28N)+EGFP (DIV 16–20) were subjected to the receptor recycling assay (see Text S1 for details). The surface-to-internal ratios of GluR2 were measured at the indicated time points after acid strip, and normalized to those of 0 min controls. n = 15–20, * p<0.05, Student's t-test. The normalized ratios of RalA WT and RalA S28N at 20 min were not significantly different (p = 0.35, Student's t-test). (B) RalA knockdown does not affect GluR2 recycling. Cultured neurons doubly transfected with HA-GluR2+sh-RalA or sh-vec (DIV 16–20) were subjected to the receptor recycling assay. n = 25–27, *** p<0.001, Student's t-test. The normalized ratios of sh-RalA and sh-vec at 20 min were not significantly different (p = 0.08, Student's t-test). (1.25 MB TIF) [file pbio.1000187.s009.tif]

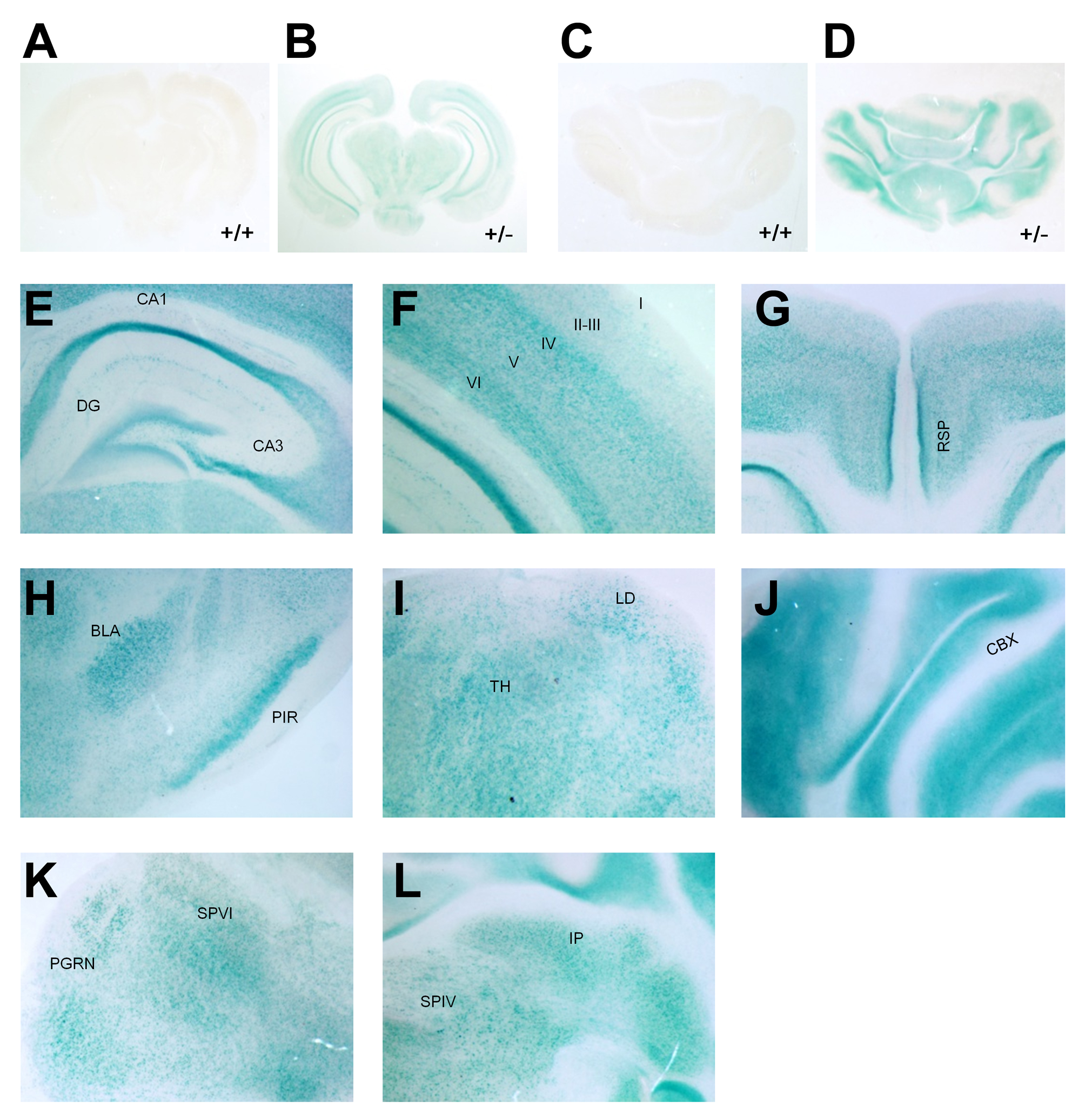

Supplement: Figure S10 — Distribution patterns of RalBP1-β-geo fusion proteins in mouse brain regions. (A–D) Widespread distribution patterns of RalBP1-β-geo fusion proteins in mouse brain regions. Forebrain (A, B) and cerebellum (C, D) sections prepared from 4-wk-old RalBP1+/+ and RalBP1+/− littermates were stained by X-gal. Note that the lack of X-gal staining in WT brain indicates the specific expression of RalBP1-β-geo fusion proteins in RalBP1+/− slices. (E–L) High-magnification images of RalBP1-β-geo fusion protein expression in RalBP1+/− brain regions including hippocampus (E), cerebral cortex (F), retrosplenial area (G), amygdala (H), thalamus (I), cerebellum (J), and brainstem (K and L). RSP, retrosplenial area; BLA, basolateral amygdalar nucleus; PIR, piriform area; LD, lateral dorsal nucleus of thalamus; TH, thalamus; PGRN, paragigantocellular reticular nucleus in the medulla; SPVI, spinal nucleus of the trigeminal in the medulla; IP, interposed nucleus in the deep cerebellar nuclei; SPIV, spinal vestibular nucleus in the junctional area between medulla and pons. (3.96 MB TIF) [file pbio.1000187.s010.tif]
