# Supplementary material for: Regulated RalBP1 Binding to RalA and PSD-95 Controls AMPA Receptor Endocytosis and LTD
Source: PLoS Biol. 2009 Sep 8;7(9):e1000187. doi: 10.1371/journal.pbio.1000187 (PMC2730530; doi:10.1371/journal.pbio.1000187)
Supplement: Text S1 — Supplementary materials and methods . (0.03 MB DOC) [file pbio.1000187.s011.doc]

**Supporting information for Han et al.,**

**“Regulated RalBP1 Binding to RalA and PSD-95 Controls** **AMPA Receptor Endocytosis and LTD”**

**Supplementary Materials and Methods**

## In Situ Hybridization Analysis

Hybridization probes specific for RalA, RalBP1, and POB1 were prepared using the following regions: nt 168-688 of rat RalA (coding region, nt 37-658), nt 1501-2067 of rat RalBP1 (coding region, nt 124-2067), and nt 503-980 of rat POB1 (XM_001072216; coding region, nt 1-2283). Antisense riboprobes were generated using α-^35^S-UTP and Riboprobe System (Promega).

#### GluR2 Recycling Assay

Live labeling of HA-GluR2 was performed as in the antibody feeding assay. After brief washing in prewarmed DMEM, neurons were returned to normal conditioned medium and incubated for 10 min at 37 ^o^C to allow endocytosis. Remaining surface HA-GluR2-bound HA antibodies were acid-stripped with 0.5 M NaCl/0.2 M acetic acid on ice for 4 min. Neurons were returned to normal conditioned medium and incubated for 20 min at 37 ^o^C to allow recycling. Neurons were fixed, and recycled receptors were labeled with Cy3-conjugated secondary antibodies. After permeabilization, internalized receptors were labeled with Cy5-conjugated secondary antibodies. Coexpressed proteins were visualized by suitable primary and FITC-conjugated secondary antibodies.

**Characterization of RalBP1 genetrap mice**

Primers for PCR genotyping were specific to RalBP1 exon 3 (5’- CCCTTGGTTGACGCAGTAGA-3’), RalBP1 intron 3 (5’- AGTAAAACCTCGCGGAAGGA-3’), and gene trap cassette (5’- CCTGGCCTCCAGACAAGTAG-3’).
